# Ras-Induced Changes in H3K27me3 Occur after Those in Transcriptional Activity

**DOI:** 10.1371/journal.pgen.1003698

**Published:** 2013-08-29

**Authors:** Masaki Hosogane, Ryo Funayama, Yuichiro Nishida, Takeshi Nagashima, Keiko Nakayama

**Affiliations:** Department of Cell Proliferation, United Center for Advanced Research and Translational Medicine, Graduate School of Medicine, Tohoku University, Seiryo-machi, Aoba-ku, Sendai, Japan; Stanford University School of Medicine, United States of America

## Abstract

Oncogenic signaling pathways regulate gene expression in part through epigenetic modification of chromatin including DNA methylation and histone modification. Trimethylation of histone H3 at lysine-27 (H3K27), which correlates with transcriptional repression, is regulated by an oncogenic form of the small GTPase Ras. Although accumulation of trimethylated H3K27 (H3K27me3) has been implicated in transcriptional regulation, it remains unclear whether Ras-induced changes in H3K27me3 are a trigger for or a consequence of changes in transcriptional activity. We have now examined the relation between H3K27 trimethylation and transcriptional regulation by Ras. Genome-wide analysis of H3K27me3 distribution and transcription at various times after expression of oncogenic Ras in mouse NIH 3T3 cells identified 115 genes for which H3K27me3 level at the gene body and transcription were both regulated by Ras. Similarly, 196 genes showed Ras-induced changes in transcription and H3K27me3 level in the region around the transcription start site. The Ras-induced changes in transcription occurred before those in H3K27me3 at the genome-wide level, a finding that was validated by analysis of individual genes. Depletion of H3K27me3 either before or after activation of Ras signaling did not affect the transcriptional regulation of these genes. Furthermore, given that H3K27me3 enrichment was dependent on Ras signaling, neither it nor transcriptional repression was maintained after inactivation of such signaling. Unexpectedly, we detected unannotated transcripts derived from intergenic regions at which the H3K27me3 level is regulated by Ras, with the changes in transcript abundance again preceding those in H3K27me3. Our results thus indicate that changes in H3K27me3 level in the gene body or in the region around the transcription start site are not a trigger for, but rather a consequence of, changes in transcriptional activity.

## Author Summary

Trimethylation of histone H3 at lysine-27 (H3K27) has been associated with silencing of gene expression. Abnormalities of this modification are thought to contribute to the epigenetic silencing of tumor suppressor genes and are regarded as a hallmark of cancer. It has remained unclear, however, whether the production of trimethylated H3K27 (H3K27me3) is the cause or the consequence of gene silencing. To address this issue, we examined the time courses of changes in H3K27me3 level and those in gene transcription induced by an oncogenic form of the Ras protein, the gene for which is one of the most frequently mutated in human cancer. We found that the amount of H3K27me3 was inversely related to transcriptional activity both at the genome-wide level and at the level of individual genes. However, we also found that the Ras-induced changes in H3K27me3 level occurred after those in transcriptional activity. Our results thus demonstrate that changes in H3K27me3 abundance are a consequence rather than a cause of transcriptional regulation, and they suggest that oncoprotein-driven changes in gene transcription can alter the pattern of histone modification in cancer cells.

## Introduction

Epigenetic modification of chromatin is a key mechanism for regulation of gene expression [1], [2]. Trimethylation of histone H3 at lysine-27 (H3K27) is associated with transcriptional repression and is regulated by Polycomb repressive complex 2 (PRC2), a histone methyltransferase specific for H3K27 [3]. This modification of H3K27 (H3K27me3) and Polycomb group proteins are thought to promote the formation of closed chromatin structures and thereby to repress transcription [4], [5]. H3K27me3 controls Hox gene silencing and X chromosome inactivation, and it is therefore essential for normal development [6], [7]. Dysregulation of H3K27me3 is also frequently observed in and is regarded as a hallmark of cancer, with global as well as site-specific increases or decreases in H3K27me3 levels having been detected in several tumor types [8]–[10].

Chromatin immunoprecipitation (ChIP) followed by deep sequencing (ChIP-seq) as well as chip-based ChIP have been applied to map precisely the distribution of H3K27me3 across the entire genome. These approaches have also been adopted to elucidate the relation between the distribution of H3K27me3 and transcriptional activity. Such studies have revealed at least two patterns of H3K27me3 enrichment associated with transcriptional repression: a focal enrichment around the transcription start site (TSS) and a broad enrichment encompassing the entire gene. H3K27me3 around the TSS frequently colocalizes with H3K4me3 and is associated with gene repression especially in undifferentiated cells [11], [12]. A broad enrichment of H3K27me3, also known as a blanket-type pattern or broad local enrichment (BLOC), has been detected over larger genomic regions including the TSS [13]–[17]. This pattern of modification has been associated not only with individual repressed genes but also with repressed gene clusters, and it is frequently observed in differentiated cells. Furthermore, both of these enrichment patterns are highly variable among cell types [18], [19], indicating that the distribution of H3K27me3 is regulated in a manner dependent on the cellular and developmental context.

The small GTPase Ras controls cell growth and survival in part through epigenetic modification including DNA methylation and histone modification. Ras regulates the activity of downstream signaling pathways including those mediated by mitogen-activated protein kinases (MAPKs) [20], [21]. The activating G12V amino acid substitution is one of the most frequent Ras mutations found in human cancer. Ras up-regulates the expression of p16*^Ink4a^*, an inhibitor of cyclin-dependent kinases, and this effect is accompanied by a marked decrease in the amount of H3K27me3 at the *Ink4a* locus in mouse embryonic fibroblasts [22]–[25]. Moreover, Ras-induced oncogenic transformation of mouse NIH 3T3 cells is associated with the down-regulation of *Fas*, *Reck*, and *Par4* transcription concomitant with an increase in DNA methylation [26]–[28].

Most of the reported associations between H3K27me3 status and transcription are based on correlation. It has thus remained to be determined definitively whether changes in H3K27me3 distribution are causal with regard to regulation of transcription. To elucidate the biological relevance of H3K27me3, we have now investigated the time courses of Ras-induced changes in H3K27me3 level and in transcription at the genome-wide level in NIH 3T3 cells. Our results indicate that changes in H3K27me3 status follow, rather than precede, transcriptional changes induced by Ras signaling.

## Results

### H3K27me3 is an epigenetic modification regulated by Ras signaling

**Figure 1:**
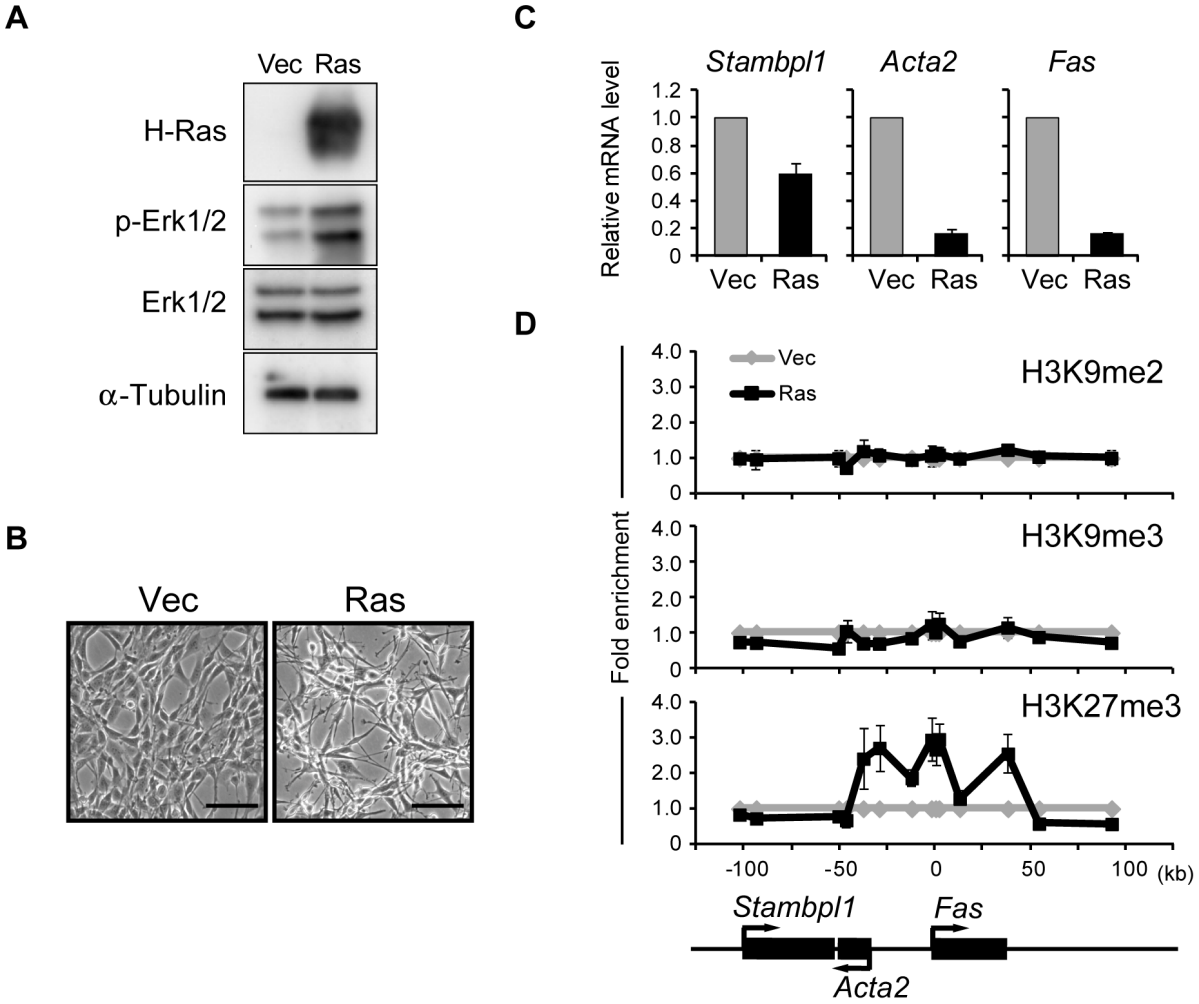
Activation of Ras signaling increases H3K27me3 abundance at the *Fas* locus in NIH 3T3 cells. (**A**) Immunoblot analysis of H-Ras, phosphorylated (p-) and total forms of Erk1/2, and α-tubulin (loading control) in the cytosolic fraction of NIH 3T3 cells expressing human H-Ras(G12V) (Ras cells) and control (Vec) cells. (**B**) Phase-contrast images of Ras and Vec cells. Scale bars, 100 µm. (**C**) RT-qPCR analysis of *Fas*, *Acta2*, and *Stambpl1* expression in Ras cells relative to that in Vec cells. Data are means ± SE from five independent experiments. (**D**) ChIP-qPCR analysis of H3K9me2, H3K9me3, and H3K27me3 at the *Fas* locus in Ras and Vec cells. The positions of genes on the chromosome and their transcriptional orientation are indicated at the bottom of the panel. Data are expressed as fold enrichment relative to the value for Vec cells at each position, and are means ± SE from at least two independent experiments.

We established mouse NIH 3T3 cells that express a constitutively active mutant (G12V) of human H-Ras or that were infected with the corresponding empty retroviral vector (referred to hereafter as Ras cells and Vec cells, respectively). Expression of the Ras transgene resulted in increased phosphorylation of the MAPK isoforms Erk1 and Erk2 (Figure 1A) as well as in morphological transformation of the cells (Figure 1B). Moreover, reverse transcription (RT) and quantitative polymerase chain reaction (qPCR) analysis revealed that the Ras cells exhibited transcriptional repression of *Fas* locus genes including *Fas*, *Acta2*, and *Stambpl1* (Figure 1C), consistent with previous observations [26], [28].

Fas is a cell surface receptor that mediates the induction of apoptosis by Fas ligand [29]. Although Ras signaling has been reported to increase the level of DNA methylation around the *Fas* locus [26], [28], we did not detect such an obvious effect (data not shown). To identify histone modifications that might contribute to silencing of the *Fas* locus, we performed ChIP-qPCR analysis with antibodies to transcriptionally repressive histone marks including H3K9me2, H3K9me3, and H3K27me3 (Figure 1D). Among these marks, only the amount of H3K27me3 was increased at the *Fas* locus of Ras cells. The H3K27me3-enriched region contained the entire *Fas* gene as well as the promoter of *Acta2*. These results thus showed that Ras signaling induces trimethylation of H3K27 as an epigenetic modification.

### Identification of H3K27me3-enriched genomic regions associated with transcriptional silencing

**Figure 2:**
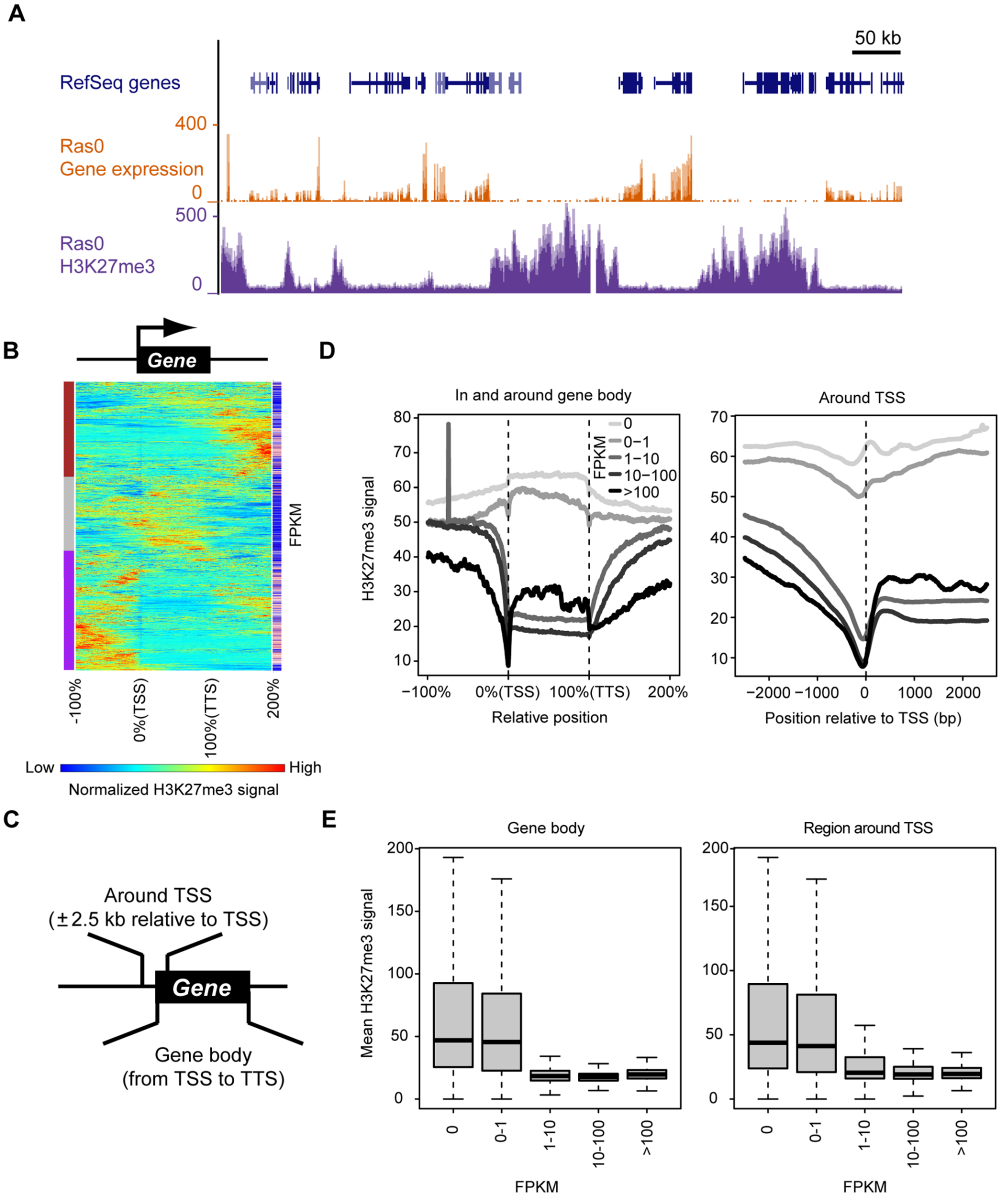
Genome-wide identification of genomic regions at which H3K27me3 enrichment is associated with transcriptional repression. (**A**) Representative distribution of gene expression level and H3K27me3 abundance (as determined by RNA-seq and ChIP-seq, respectively) in control (Ras0) cells. Both parameters are normalized by total read counts. (**B**) Clustering of 2000 randomly selected genes of control cells based on H3K27me3 level. Each line represents an individual gene, including the upstream region, gene body, and downstream region. The length of the gene body is defined as 100% (consisting of 200 data points), and the flanking regions are ±100% of the gene body. Results of hierarchical clustering are depicted on the left with colors of brown, gray, and purple. The expression level (FPKM) for individual genes is depicted on the right with colors from blue (low FPKM) to red (high FPKM). (**C**) Definition of the gene body and the region around the TSS for the purposes of this study. (**D**) Relation between gene expression (FPKM) and H3K27me3 level both in and around the gene body (left) and in the region around the TSS (right) for all RefSeq genes. (**E**) Relation between gene expression (FPKM) and mean H3K27me3 level either in the gene body (left) or in the region around the TSS (right) for all RefSeq genes. The plots show the median, 25th and 75th percentiles, and range.

To determine whether Ras-induced changes in H3K27me3 abundance are a trigger for or a consequence of changes in transcription, we set out to analyze the time courses of these events at the genome-wide level in NIH 3T3 cells infected with the retrovirus for H-Ras(G12V) at time 0. Transcript and H3K27me3 levels were measured by RNA-seq and ChIP-seq, respectively (detailed sequencing information is provided in Table S1). First, we identified regions of H3K27me3 enrichment associated with silent genes in cells before introduction of H-Ras(G12V) (Ras0 cells) (Figure 2A). H3K27me3 showed broad enrichment domains encompassing several hundred kilobases, consistent with previous observations [13], [15]. To characterize the pattern of H3K27me3 within genes, we divided each gene into the gene body, upstream region, and downstream region, with gene body being defined as the genomic region from the TSS to the transcription termination site (TTS). From a total of 23,232 RefSeq genes, we randomly selected 2000 genes and ordered them according to similarity in the pattern of H3K27me3 enrichment (Figure 2B). This analysis revealed that the pattern of H3K27me3 enrichment fell into three distinct clusters (designated brown, gray, and purple clusters). In the gray cluster, H3K27me3 covered the gene body as well as the region around the TSS. This cluster contained a high proportion of transcriptionally repressed genes, as represented by the bluish color in the FPKM (fragments per kilobase of exon model per million mapped fragments) column. This finding was confirmed by a different method examining all RefSeq genes, as detailed below. We next focused on the H3K27me3 signal in the gene body or in the region around the TSS of each gene (Figure 2C). RefSeq genes were classified into five groups according to their expression level. In the groups containing repressed genes (FPKM of 0 or 0–1), H3K27me3 was localized to the gene body as well as to the region around the TSS (Figure 2D). In contrast, in the groups containing expressed genes (FPKM of 1–10, 10–100, or >100), H3K27me3 was present at a low level in the gene body and in the nucleosome-free region around the TSS. The mean H3K27me3 signals in the gene body and in the region around the TSS of each gene also reflected the transcriptional status of the corresponding genes (Figure 2E). These data indicated that enrichment of H3K27me3 in the gene body as well as in the region around the TSS reflects silenced transcription.

### Comprehensive analysis of Ras-induced changes in transcription and H3K27me3 content

**Figure 3:**
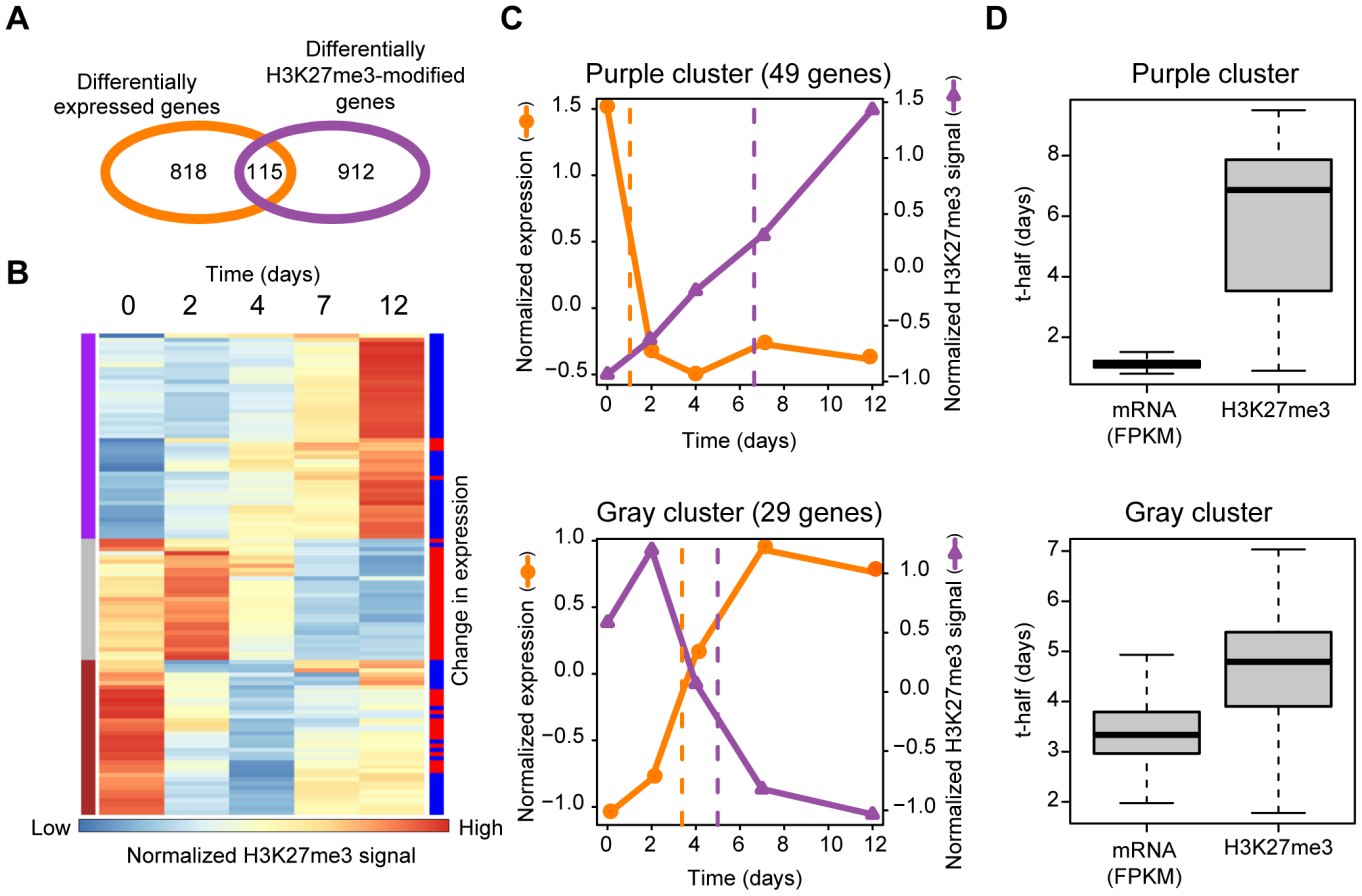
Comprehensive analysis of Ras-induced changes in gene transcription and H3K27me3 content in the gene body. (**A**) Venn diagram indicating the number of genes showing Ras-induced changes in expression and in the mean H3K27me3 level of the gene body. (**B**) Clustering of the temporal profiles of mean H3K27me3 level in the gene body. Each line represents one of 115 genes whose H3K27me3 level in the gene body and expression changed in NIH 3T3 cells during expression of H-Ras(G12V) for the indicated times. Results of hierarchical clustering are depicted on the left with colors of purple, gray, and brown. Changes in expression level (FPKM) of individual genes (as determined in Figure S1B) are depicted on the right with colors of red (increase) or blue (decrease). (**C**) Averaged changes in expression and H3K27me3 level for the purple cluster (upper) and the gray cluster (lower) of genes shown in (B). Dashed lines represent “t-half,” the time corresponding to half of the difference between the values for Ras0 cells and cells expressing H-Ras(G12V) for 12 days. (**D**) The t-half values for expression and mean H3K27me3 level in the gene body for the purple and the gray clusters in (B).

We next identified genes whose transcription and H3K27me3 level are both regulated by Ras. We calculated the fold change in mean H3K27me3 level over the gene body for individual genes in cells infected with the Ras retroviral vector for 2, 4, 7, or 12 days relative to that in Ras0 cells. Among a total of 23,232 RefSeq genes, 1027 genes showed at least a twofold change in H3K27me3 level at least one time point (Figure 3A). A total of 933 genes showed a significant change in expression level at at least one time point after Ras introduction (see Materials and Methods). We then subjected the 115 genes whose H3K27me3 level and expression were both regulated by Ras to hierarchical clustering based on the time course of the change in H3K27me3 abundance (Figure 3B). This analysis revealed three distinct patterns of H3K27me3 dynamics induced by Ras: A purple cluster of genes in which the H3K27me3 level increased after Ras activation, and gray and brown clusters in which the H3K27me3 level decreased. Whereas changes in H3K27me3 abundance in the brown cluster were not associated with a characteristic transcriptional trend, those in the purple and gray clusters were inversely correlated with changes in transcription (Figure 3B, Figure S1A and S1B). Moreover, in these transcription-correlated clusters, changes in transcription were apparent within 2 days after Ras activation, whereas the mean H3K27me3 level remained essentially unchanged at this time point (Figure 3C). We calculated “t-half” to evaluate the timing of these two events (Figure S1C). In the purple cluster, the median t-half for mRNA abundance occurred at 1.1 days and that for H3K27me3 level occurred at 6.9 days (Figure 3D). In the gray cluster, the median t-half for mRNA abundance occurred at 3.3 days and that for H3K27me3 level occurred at 4.8 days. These results thus indicated that changes in transcription precede those in H3K27me3 level in the gene body.

We performed a similar analysis for the 196 genes whose H3K27me3 level in the region around the TSS and expression were both regulated by Ras (Figure S2). Similar to the case for H3K27me3 in the gene body, increases in H3K27me3 level in the region around the TSS occurred after decreases in transcription. Together, our genome-wide comprehensive analyses thus revealed that Ras signaling affects transcription before it affects mean H3K27me3 level both in the gene body and in the region around the TSS.

### Characterization of genes whose H3K27me3 level is altered after transcriptional changes induced by Ras signaling

**Figure 4:**
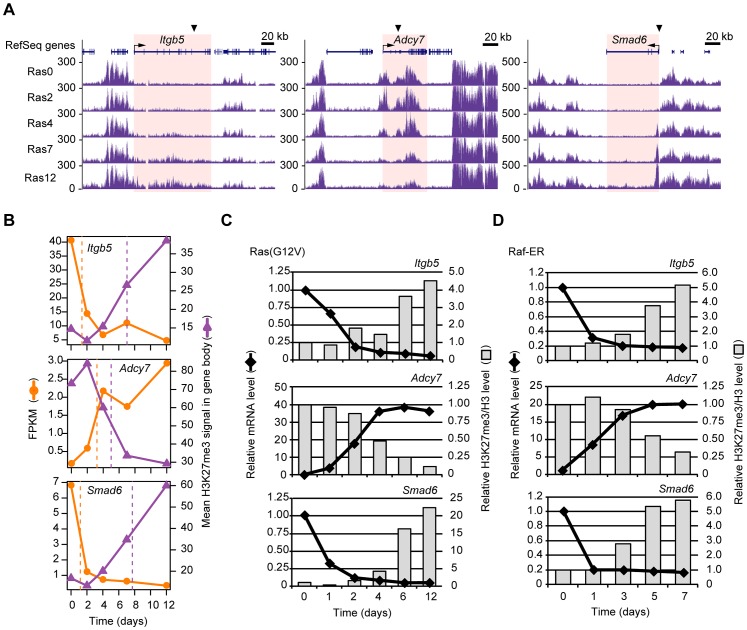
Validation of the temporal sequence of changes in gene expression and H3K27me3 level induced by Ras signaling. (**A**) Time course of changes in H3K27me3 level at the *Itgb5*, *Adcy7*, and *Smad6* loci as determined by ChIP-seq analysis of Ras0 cells and cells infected with the retroviral vector for H-Ras(G12V) for 2, 4, 7, or 12 days. The regions for which the mean H3K27me3 level and corresponding t-half were calculated are highlighted in pink. (**B**) Changes in gene expression (FPKM) and mean H3K27me3 level for *Itgb5*, *Adcy7*, and *Smad6*. The t-half values are indicated by the dashed lines. (**C**) RT-qPCR analysis of gene expression and ChIP-qPCR analysis of the ratio of H3K27me3 to total H3 for *Itgb5*, *Adcy7*, and *Smad6* at the indicated times after introduction of the retroviral vector for H-Ras(G12V). Data are expressed relative to the corresponding value for time 0. The positions of PCR primers are indicated by arrowheads in (A), and correspond to positions e for *Itgb5* and i for *Adcy7* shown in Figure S4. Data are representative of four independent experiments. (**D**) Gene expression (RT-qPCR) and the ratio of H3K27me3 to total H3 (ChIP-qPCR) at the indicated times after exposure of NIH 3T3 cells expressing Raf-ER to 4HT. Data are representative of four independent experiments.

We selected three gene loci—*Itgb5*, *Adcy7*, and *Smad6*—for further study to confirm the results of our genome-wide RNA-seq and ChIP-seq analyses. *Itgb5* and *Adcy7* manifested Ras-induced changes in H3K27me3 level in the gene body (Figure 4A). The time courses of the ChIP-seq and RNA-seq data showed that Ras signaling initially affected transcription and then gradually changed the H3K27me3 content of the gene body for *Itgb5* and *Adcy7* (Figure 4B) as well as for four additional genes, *Plekha4*, *Ephx1*, *Bpifc*, and *Sorcs2* (Figure S3). In the case of *Smad6*, the H3K27me3 level increased prominently in the region around the TSS but only slightly in the gene body as previously reported (Figure 4A) [30]. Ras signaling again affected transcription first and then gradually changing H3K27me3 content (Figure 4B). In addition to *Smad6*, we found other genes that showed a prominent increase in H3K27me3 level in the region around the TSS by visual inspection of the genome browser (data not shown). These results for *Itgb5*, *Adcy7*, and *Smad6* were confirmed by RT-qPCR and ChIP-qPCR analyses (Figure 4C). We also confirmed that changes in gene expression precede those in H3K27me3 level with the use of NIH 3T3 cells that stably express Raf-ER, a fusion protein composed of the catalytic domain of Raf-1 and the ligand binding domain of the estrogen receptor. Treatment of these cells with 4-hydroxytamoxifen (4HT) activates Raf-ER and downstream MAPK pathways [31]. Activation of Raf-ER thus also affected mRNA abundance before H3K27me3 level for *Itgb5*, *Adcy7*, and *Smad6* (Figure 4D) as well as for four additional genes, *Plekha4*, *Ephx1*, *Bpifc*, and *Sorcs2* (Figure S7A).

We also evaluated the Ras-induced changes in transcription and H3K27me3 level in the gene body for *Itgb5* and *Adcy7* by independent deep sequencing and qPCR with several primer sets (Figure S4), again confirming our results. Total histone H3 level in the gene body of *Itgb5* or *Adcy7* was affected only slightly by Ras signaling (Figure S4D and S4H). The altered H3K27me3 content of the gene body was thus likely due to a change in H3K27 trimethylation, not to a change in nucleosome density. In addition to H3K27me3, we also examined H3K9me2 and H3K9me3 levels (Figure S5). Among these repressive histone marks, only H3K27me3 was markedly altered by Ras signaling. Together, these data suggested that our genome-wide analyses correctly identified genes that undergo changes in transcription and H3K27me3 level in response to Ras signaling, and they confirmed that the changes in transcription precede those in H3K27me3 level.

### Changes in H3K27me3 level are a consequence of those in Ras-induced transcription

**Figure 5:**
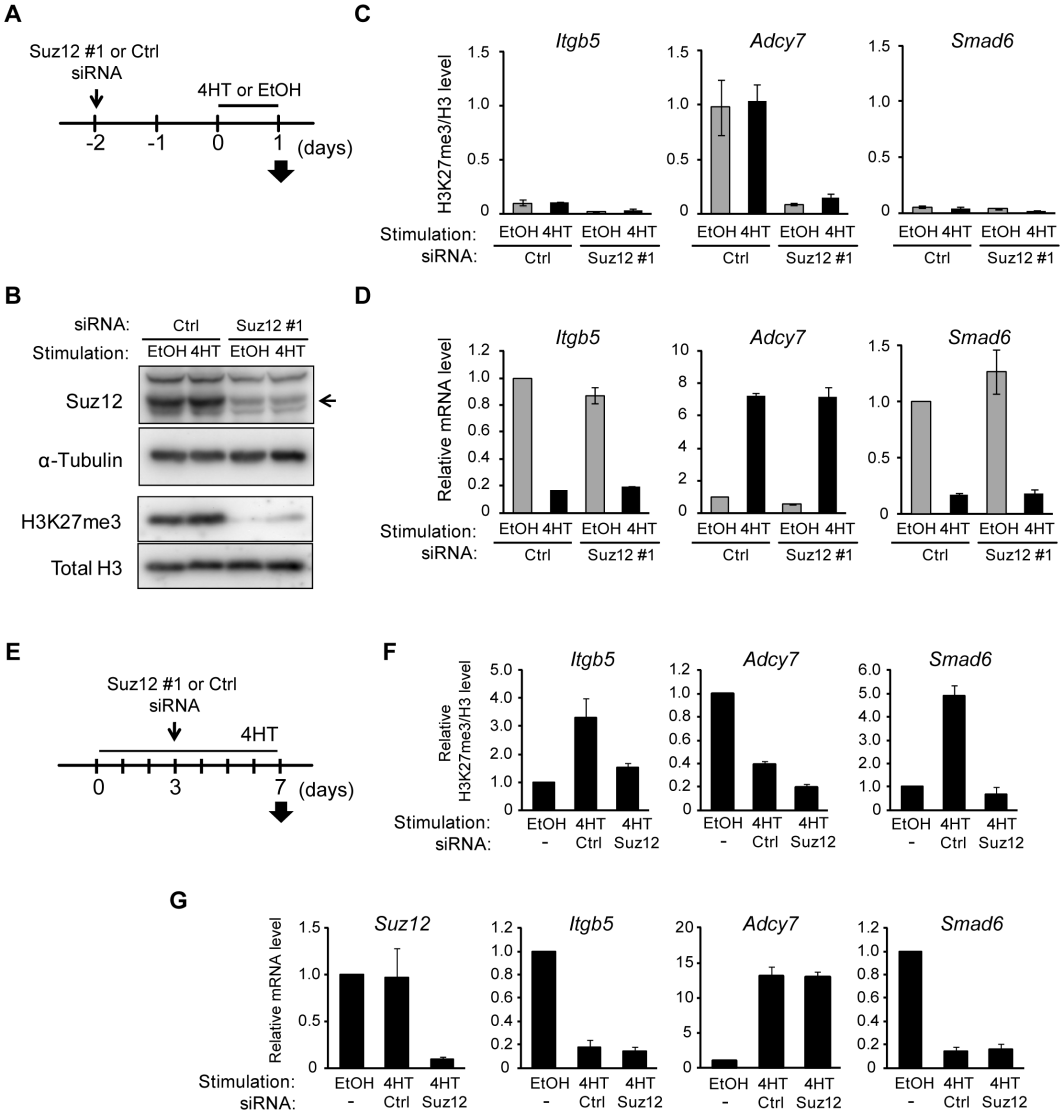
Signaling-induced changes in H3K27me3 level are not required for those in transcriptional activity. (**A**) Time line for transfection with control (Ctrl) or Suz12 siRNAs, treatment with 4HT or ethanol (EtOH) vehicle, and sample analysis (arrow) for NIH 3T3–Raf-ER cells studied in (B) through (D). (**B**) Immunoblot analysis of Suz12 (arrow) and α-tubulin in the cytosolic fraction as well as of H3K27me3 and total H3 in the chromatin fraction. (**C**) ChIP-qPCR analysis of H3K27me3 normalized by total H3 for the regions of *Itgb5*, *Adcy7*, and *Smad6* indicated in Figure 4A. Data are means ± SE from two independent experiments. (**D**) RT-qPCR analysis of relative *Itgb5*, *Adcy7*, and *Smad6* expression. Data are means ± SE from three independent experiments. (**E**) Time line for transfection with control or Suz12 siRNAs, treatment with 4HT or ethanol vehicle, and sample analysis (arrow) for NIH 3T3–Raf-ER cells studied in (F) and (G). (**F**) ChIP-qPCR analysis of H3K27me3 normalized by total H3 at *Itgb5*, *Adcy7*, and *Smad6*. Data are means ± SE from two independent experiments. (**G**) RT-qPCR analysis of relative *Suz12*, *Itgb5*, *Adcy7*, and *Smad6* expression. Data are means ± SE from two independent experiments.

Our results suggested that a change in the amount of H3K27me3 is not required for Ras-induced regulation of gene transcription. To verify this hypothesis, we prepared NIH 3T3–Raf-ER cells depleted of H3K27me3 by transfection with small interfering RNAs (siRNAs) for Suz12, a subunit of PRC2 that is indispensable for methyltransferase activity at H3K27 [32]. The cells were transfected with Suz12 siRNA for 48 h before exposure to 4HT for 24 h (Figure 5A), and they were then analyzed for effects on H3K27me3 and transcription. Immunoblot and ChIP-qPCR analyses revealed that knockdown of Suz12 resulted in depletion of H3K27me3 in the total chromatin fraction (Figure 5B) as well as at specific regions such as *Itgb5*, *Adcy7*, and *Smad6* loci (Figure 5C, Figure S6A–S6C). Depletion of H3K27me3 did not affect the 4HT-induced repression of *Itgb5* and *Smad6* expression (Figure 5D), indicating that an increase in the level of H3K27me3 is not required for Ras-induced transcriptional silencing of these genes. Furthermore, H3K27me3 depletion did not induce expression of *Adcy7* in the absence of 4HT (Figure 5D), indicating that depletion of H3K27me3 is not sufficient to induce transcriptional activation. We obtained similar results with two additional Suz12 siRNAs (Figure S6D–S6F) and four additional genes, *Plekha4*, *Ephx1*, *Bpifc*, and *Sorcs2* (Figure S7).

We also examined the effect of H3K27me3 depletion after the activation of Raf signaling by transfecting NIH 3T3–Raf-ER cells with Suz12 siRNA 3 days after exposure to 4HT (Figure 5E and 5G). Analysis of the cells at 7 days after the onset of Raf activation revealed that Suz12 siRNA efficiently suppressed the increase in H3K27me3 level at *Itgb5* and *Smad6* (Figure 5F). Nevertheless, this effect did not induce expression of *Itgb5* and *Smad6* (Figure 5G), indicating that depletion of H3K27me3 does not affect transcriptional suppression of *Itgb5* and *Smad6* by Ras signaling. These data suggested that changes in H3K27me3 abundance do not play a critical role in the induction of gene silencing at later stages of Ras activation.

Together, our observations indicated that a change in the level of H3K27me3 induced by Ras is not a trigger for, but rather a consequence of, a change in transcription.

### Accumulation of H3K27me3 is dependent on Ras signaling and reversible

**Figure 6:**
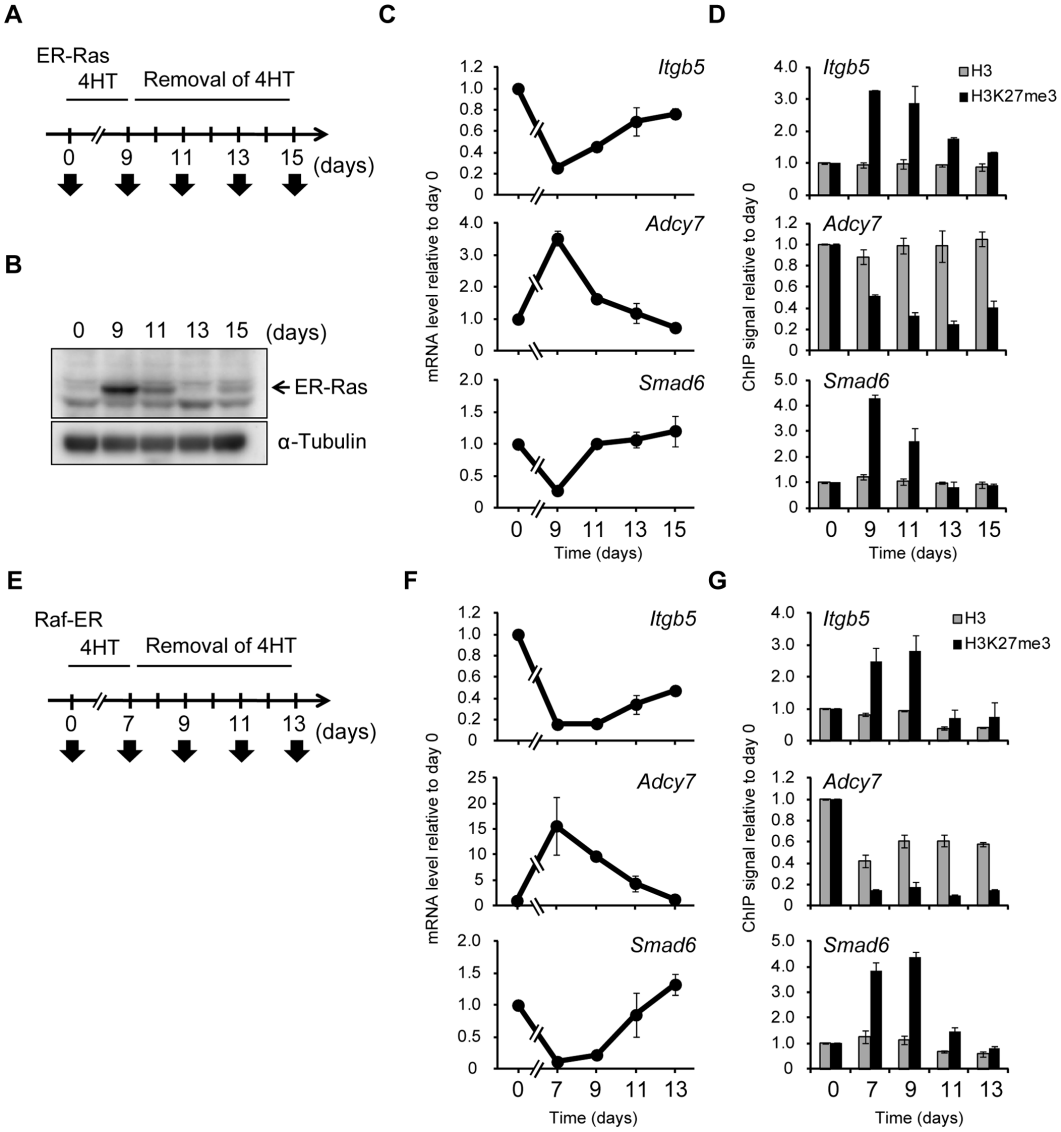
Ras-induced H3K27me3 accumulation and transcriptional changes are reversed by inactivation of Ras signaling. (**A**) Time line for exposure to and removal of 4HT as well as sample analysis (arrows) for NIH 3T3–ER-Ras cells studied in (B) through (D). (**B**) Immunoblot analysis of ER-Ras (arrow) and α-tubulin in the cytosolic fraction of the cells. (**C**) RT-qPCR analysis of relative *Itgb5*, *Adcy7*, and *Smad6* expression. Data are means ± SE from two independent experiments. (**D**) ChIP-qPCR analysis of H3K27me3 and total H3 levels for the regions of *Itgb5*, *Adcy7*, and *Smad6* indicated in Figure 4A. Data are means ± SE from two independent experiments. (**E**) Time line for exposure to and removal of 4HT as well as sample analysis (arrows) for NIH 3T3–Raf-ER cells studied in (F) and (G). (**F**) RT-qPCR analysis of relative *Itgb5*, *Adcy7*, and *Smad6* expression. Data are means ± SE from two independent experiments. (**G**) ChIP-qPCR analysis of H3K27me3 and total H3 at *Itgb5*, *Adcy7*, and *Smad6*. Data are means ± SE from two independent experiments. The position of PCR primers of *Itgb5* correspond to positions c in Figure S4A.

The presence of H3K27me3 at an exogenous transgene was previously shown to maintain the repressed state [33], suggesting the possibility that an increase in H3K27me3 level induced by Ras signaling might be able to maintain repression of gene expression after signaling is inactivated. To test this possibility, we introduced ER-Ras [a fusion protein of human H-Ras(G12V) and the estrogen receptor] into NIH 3T3 cells, exposed the cells to 4HT for 9 days in order to induce changes in both H3K27me3 level and transcription, and then removed 4HT to inactivate Ras signaling (Figure 6A). Immunoblot analysis revealed that ER-Ras was induced by 4HT and that its abundance decreased rapidly after removal of 4HT (Figure 6B), the latter indicative of inactivation of the Ras signal. Changes in the transcription of *Itgb5*, *Adcy7*, and *Smad6* were also apparent after exposure of the cells to 4HT for 9 days, whereas these changes were completely reversed after 4HT removal (Figure 6C). Moreover, an increase in H3K27me3 content at *Itgb5* and *Smad6* was observed in the presence of 4HT, whereas H3K27me3 abundance at these genes returned essentially to basal levels after signal inactivation (Figure 6D). The H3K27me3 level at *Adcy7* was reduced by exposure of the cells to 4HT and remained low after 4HT removal, suggesting that the dynamics of H3K27 methylation and demethylation might differ. We obtained similar results with four additional genes—*Plekha4*, *Ephx1*, *Bpifc*, and *Sorcs2* (Figure S8)—as well as with cells expressing Raf-ER (Figure 6E–6G). From these data, we concluded that changes in H3K27me3 level are dependent on Ras signaling, and that H3K27me3 enrichment is not maintained after inactivation of such signaling, resulting in reactivation of transcription.

### Changes in H3K27me3 content in intergenic regions predict the presence of unannotated transcripts

**Figure 7:**
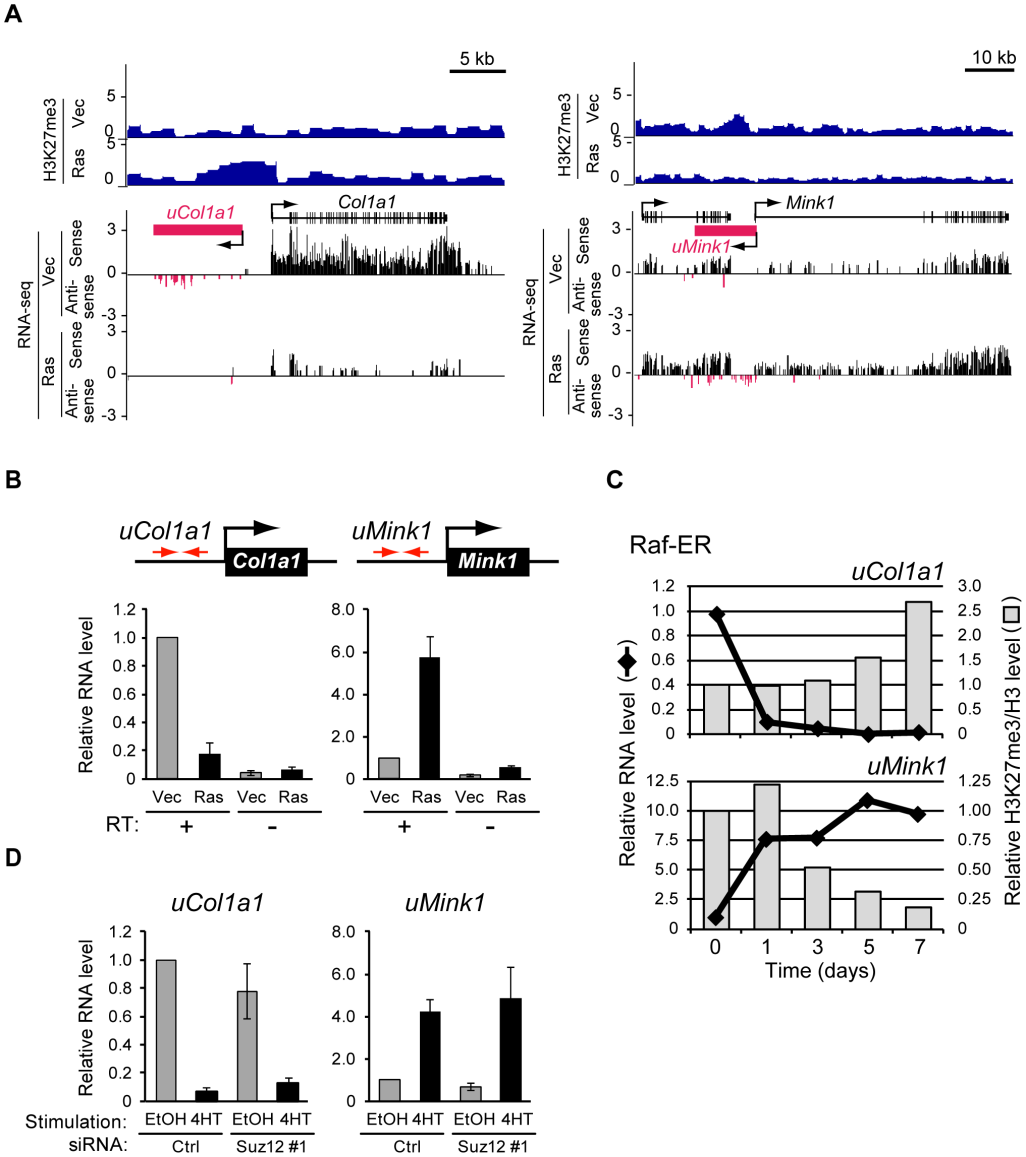
Signaling-induced changes in the production of novel transcripts from intergenic regions occur before changes in H3K27me3 level. (**A**) ChIP-seq analysis of H3K27me3 level as well as strand-specific assignment of sequencing reads from RNA-seq analysis by SOLiD sequencing for *Col1a1* and *Mink1* loci in Ras and Vec cells. Antisense transcription from the region upstream of each gene was detected predominantly in the vicinity of regions that showed changes in H3K27me3 level, with the predicted transcribed region being denoted schematically by the magenta box. (**B**) RT-qPCR analysis of transcripts derived from the regions upstream of *Col1a1* (*uCol1a1*) and *Mink1* (*uMink1*) in Ras and Vec cells. The analysis was performed with or without the RT reaction. Primers (red arrows) were targeted to the intergenic regions sensitive to Ras-induced modulation of H3K27me3 content. Data are means ± SE from four independent experiments. (**C**) RT-qPCR analysis of expression as well as ChIP-qPCR analysis of H3K27me3 normalized by total H3 for *uCol1a1* and *uMink1* at the indicated times after exposure of NIH 3T3 cells expressing Raf-ER to 4HT. Data are expressed relative to the values for time 0 and are representative of four independent experiments. (**D**) RT-qPCR analysis of the relative abundance of transcripts derived from *uCol1a1* and *uMink1* in NIH 3T3–Raf-ER cells transfected with Suz12 or control siRNAs and exposed to 4HT or ethanol as in Figure 5A. Data are means ± SE from two independent experiments.

Visual inspection of H3K27me3 distribution revealed that Ras signaling alters H3K27me3 levels in intergenic regions located several kilobases distant from known gene bodies. Two representative loci, *Col1a1* and *Mink1*, are shown in Figure 7A. H3K27me3 was enriched in the region upstream of *Col1a1* but was depleted in the region upstream of *Mink1* in Ras cells.

Given that changes in H3K27me3 level were frequently observed in the transcribed region of genes such as *Itgb5* and *Adcy7*, we examined whether unannotated transcripts might be produced from the regions upstream of *Col1a1* and *Mink1*. We reanalyzed RNA-seq data obtained by SOLiD sequencing, which contain strand information (see Materials and Methods), and we indeed detected sequence reads for these regions, suggesting the existence of corresponding transcripts (Figure 7A). RT-qPCR analysis confirmed the presence of transcripts derived from the regions upstream of *Col1a1* and *Mink1* (hereafter referred to as *uCol1a1* and *uMink1*, respectively) (Figure 7B). Ras signaling repressed *uCol1a1* expression and activated *uMink1* expression, similar to its effects on *Col1a1* and *Mink1* mRNA levels (Figure 7A). These results thus revealed that changes in the H3K27me3 content of intergenic regions can predict the presence of unannotated transcripts.

To determine whether the changes in *uCol1a1* and *uMink1* transcription also precede those in H3K27me3 level, we examined the respective time courses with cells expressing Raf-ER (Figure 7C). The expression of *uCol1a1* and *uMink1* was altered already at 1 day after exposure of the cells to 4HT, whereas H3K27me3 level remained essentially unaffected at this time. The level of H3K27me3 changed at 5 days (*uCol1a1*) or 3 days (*uMink1*) after 4HT exposure. We obtained similar results for transcripts derived from another intergenic region, *uIl33* (Figure S9). Ras-induced transcription from intergenic regions thus also occurs prior to changes in H3K27me3 level.

To examine whether H3K27me3 is required for regulation of *uCol1a1* and *uMink1* transcription, we determined the effect of Suz12 knockdown with siRNAs. Depletion of H3K27me3 did not affect the 4HT-induced silencing of *uCol1a1*, nor did it induce *uMink1* expression in the absence of 4HT (Figure 7D). Together, these results suggested that the observed changes in H3K27me3 level in transcribed regions result from changes in transcription.

## Discussion

Many genome-wide analyses have claimed that H3K27me3 is an indicator of transcription in diverse cell lines [34], [35]. However, the lack of information about the dynamics of histone modification has left it unclear whether the level of H3K27me3 actually regulates transcriptional activity.

We have now performed RNA-seq and H3K27me3 ChIP-seq in cells at various times after the onset of expression of a constitutively active form of Ras. For RefSeq genes, we found that a Ras-induced change in transcriptional activity was inversely correlated with a change in H3K27me3 level at the gene body and in the region around the TSS. However, genome-wide analysis of the time courses of the changes in H3K27me3 abundance and transcription revealed that Ras-induced changes in transcription occurred before those in H3K27me3 level. This finding was confirmed by qPCR analysis. Furthermore, depletion of H3K27me3 with the use of siRNAs targeted to PRC2 did not affect Ras-induced transcriptional changes. We therefore conclude that Ras-induced changes in H3K27me3 level are not a trigger for, but rather a consequence of, changes in transcription.

We also found that intergenic regions that show a change in H3K27me3 content in response to Ras signaling generate unannotated transcripts. Again, this transcription preceded the change in H3K27me3 level.

### Comprehensive analysis based on the mean H3K27me3 level in defined regions

H3K27me3 has been found to manifest at least two distinct enrichment patterns—being abundant in narrow regions around the TSS and in broad domains that include entire genes—and the appearance rate of these patterns differs among cell types [18]. We have now analyzed these patterns in control NIH 3T3 (Ras0) cells. For a simple comparison of H3K27me3 level with transcription in cells at various times after the onset of Ras expression, we used the mean value of H3K27me3 level in a defined region such as the gene body or the region around (±2.5 kb) the TSS to represent the H3K27me3 status of each gene (Figure 2C). We found that enrichment of H3K27me3 not only in the region around the TSS but also in the gene body correlated inversely with transcriptional level in these cells, consistent with previous observations [18]. Comparison of the time courses of mean H3K27me3 level and transcription allowed us to identify genes for which H3K27me3 content changes together with transcriptional activity in response to Ras signaling, suggesting that the mean value of H3K27me3 level in the defined regions provides an indication of H3K27me3 status of individual genes under different cellular conditions.

We noticed by visual inspection the existence of several patterns of H3K27me3 modification within the defined regions. Although the use of mean values of H3K27me3 level disregarded these patterns, we conclude that such mean values provide a relatively simple measure for comparison of H3K27me3 status with transcriptional activity. For example, *Itgb5* manifested a typical broad increase in H3K27me3 level, whereas *Adcy*7 showed two discontinuous regions of H3K27me3 enrichment in the gene body that were depleted in parallel in response to Ras signaling, and *Smad6* exhibited a prominent increase in H3K27me3 around the TSS (Figure 4A). Although various internal patterns of H3K27me3 were observed, however, visual inspection revealed that the time courses of H3K27me3 level at each position in the defined regions were similar to those for the mean value (Figure 4A and 4B), indicating that changes in mean H3K27me3 level in the defined regions also represent changes in H3K27me3 status of genes despite differences in the internal patterns within the defined regions. Our approach based on mean H3K27me3 level in defined regions thus allows evaluation of the timing of changes in H3K27me3 abundance relative to those in transcription, and it leads us to the conclusion that Ras-induced changes in H3K27me3 level occur after those in transcription.

Our H3K27me3 ChIP-seq data contain time course information as well as higher positional resolution compared with previously published H3K27me3 ChIP-seq results [11], [15], [30]. Our data are thus amenable to analysis of other aspects of H3K27 trimethylation. For example, temporal analysis of H3K27me3 distribution at base-pair resolution might allow the unveiling of Polycomb response elements (PREs), for which little information is currently available in mammal [36]–[38].

### Causal relation between transcription and H3K27me3 status

Our results show that the Ras signaling–induced changes in transcription precede those in H3K27me3 level. Previous studies have also shown that transcriptional regulation is initiated before changes in H3K27me3 content [39]–[41]. We further revealed that an increase in H3K27me3 level induced by Ras is insufficient for maintenance of transcriptional repression after inactivation of Ras signaling. Such increases in H3K27me3 level induced by Ras signaling were thus found to be completely reversed after signal inactivation. Similar reversibility of changes in H3K27me3 level has been described for the *FLC* gene in *Arabidopsis* [41], for which transcription and H3K27me3 content are regulated by signaling that is responsive to changes in temperature. It is thus possible that a signal-induced increase in H3K27me3 abundance is dispensable for both initiation and maintenance of transcriptional repression in various cell types and different species.

On the other hand, H3K27me3 has been reported to contribute to maintenance of transcriptional suppression in other experimental systems [33], [42]. The combination of H3K27me3 with other epigenetic marks has also been found to be related to transcriptional repression [6], suggesting the possibility that Ras might regulate only H3K27me3, and not other histone marks required for maintenance of gene silencing. One such possible histone modification is ubiquitylation of histone H2A at lysine-119 [43]. H3K27me3 recruits PRC1, which functions as a ubiquitin ligase for this residue of H2A. Ubiquitylation of H2A by PRC1 results in repression of transcription by blocking the release of RNA polymerase II from promoters [44]. Not all genomic regions that show H3K27me3 enrichment colocalize with ubiquitylated H2A (H2Aub) or PRC1 [45], [46], however, suggesting that both H3K27me3 and H2Aub may be required for maintenance of gene silencing. Given that Ras-induced changes in H3K27me3 level are a consequence of those in transcription, Ras might influence H3K27me3 content without affecting H2Aub level. Further analysis of H2Aub level during Ras activation may provide insight into the function of H3K27me3.

### The mechanism of changes in H3K27me3 level and transcription induced by Ras signaling

The mechanism by which Ras signaling regulates H3K27me3 level in NIH 3T3 cells remains unclear. Changes in histone modification are mediated by changes in the expression or localization of the corresponding enzymes [47]. Changes in the expression level of enzymes have thus been found to be responsible for changes in H3K27me3 level in response to Ras signaling [24], [25]. We found that the expression of genes encoding subunits of PRC2 or PRC1 was not altered by Ras activation in NIH 3T3 cells, however (Figure S10A and S10B). Of genes for two known demethylases, the expression of only *Jmjd3* was found to be up-regulated by Ras signaling, consistent with previous observations [24], [25]. However, knockdown of *Jmjd3* expression did not affect the expression of *Adcy7* in the absence or presence of Ras signaling (Figure S10C), suggesting that the change in *Jmjd3* expression level is not required for Ras-induced changes in transcription.

Phosphorylation of several sites of Ezh2 by various kinases has been shown to alter the localization of PRC2 [48], [49]. Moreover, Msk1 and Msk2, which are downstream kinases of Ras phosphorylate serine-28 of histone H3 (a residue adjacent to K27) and this phosphorylation prevents PRC2 from recognizing H3K27 and results in passive H3K27me3 demethylation during subsequent progression of the cell cycle [50], [51]. Such phosphorylation might contribute to the regulation of H3K27me3 level by Ras in our system. It is also possible that RNA polymerase II actively erases H3K27me3 by recruiting an H3K27me3 demethylase to the transcribed region, as previously proposed [39]. In support of this idea, we found that the demethylated regions partially coincide with the gene body, along which RNA polymerase II moves. Moreover, we detected unannotated transcripts derived from intergenic regions whose H3K27me3 level is regulated by Ras. These findings indicate that transcription might trigger H3K27me3 regulation and determine the localization of H3K27me3 demethylases. Detailed analysis of H3 modification and the localization of these enzymes may provide insight into the mechanisms determining the specificity of genomic regions subject to changes in H3K27me3 level.

We have found that Ras-induced changes in transcription precede those in H3K27me3 level, suggesting that transcriptional regulation by Ras is initiated by a mechanism independent of H3K27me3. We also performed ChIP-qPCR analysis of active histone modifications and observed changes in acetylation of H3 that were coincident with initiation of transcriptional changes at 2 days after Ras induction (data not shown). Removal of active histone marks by Ras is thus a possible mechanism for Ras-mediated gene silencing. In this case, the repressive H3K27me3 mark might be deposited passively on repressed genes as a result of the loss of acetylation. Further genome-wide and time course analyses of histone acetylation are required to examine this possibility.

## Materials and Methods

### Cells, culture conditions, retrovirus infection, and 4HT treatment

NIH 3T3 cells were obtained from American Type Culture Collection (CRL-1658) and were cultured in Dulbecco's modified Eagle's medium supplemented with 10% fetal bovine serum, 1% penicillin-streptomycin, 2 mM l-glutamine, 1% MEM–non essential amino acids, and 1% sodium pyruvate (all from Life Technologies, Foster City, CA).

Complementary DNAs encoding human H-Ras(G12V) or a fusion protein of human Raf-1 and the estrogen receptor (Raf-ER) were subcloned into the retroviral vector pMX-puro [52]. A pLNCX2 vector encoding a fusion protein of the estrogen receptor and human H-Ras(G12V) (ER-Ras) was kindly provided by M. Narita [53]. These vectors were introduced into Plat-E packaging cells by transfection with use of the FuGENE6 reagent (Promega, Madison, WI). Culture supernatants containing recombinant ecotropic retroviruses were harvested for infection of proliferating NIH 3T3 cells in the presence of polybrene. The infected cells were then subjected to selection in medium containing puromycin for pMX-puro or G418 for pLNCX2. Activation of Raf-ER or ER-Ras was induced by exposure of cells to 10 or 100 nM 4HT (Sigma, St. Louis, MO), respectively, that had been dissolved in ethanol; the medium supplemented with 4HT was refreshed every day.

### RNA interference

Cells were transfected with Suz12, Bmi1, Jmjd3, or control Stealth RNAi duplexes (Life Technologies) with the use of a Neon Transfection System (Life Technologies). The Suz12 siRNA sequences are 5′-UAAAUUCUCUUCUUCCUGGACGAGU-3′, 5′-UUUGAUUGAGGUCAGGAUUCAAAGG-3′, and 5′-UAUCGUUGGUUUCUCCUGUCCAUCG-3′ for #1, #2, and #3, respectively. The Bmi1 siRNA sequence is 5′-CGUCAUGUAUGAAGAGGAACCUUUA-3′, and the Jmjd3 siRNA sequence is 5′-GGAUGACCUCUAUGCGUCCAAUAUU-3′.

### Immunoblot analysis

Cells were lysed in a solution containing 50 mM Tris-HCl (pH 7.6), 300 mM NaCl, 0.5% Triton X-100, aprotinin (10 µg/ml), leupeptin (10 µg/ml), 1 mM phenylmethylsulfonyl fluoride, 400 µM Na_3_VO_4_, 400 µM EDTA, 10 mM NaF, and 10 mM sodium pyrophosphate. The lysate was centrifuged at 20,000× *g* for 10 min at 4°C, and the resulting supernatant was isolated as a cytosolic fraction. The pellet was resuspended in lysis solution for use as a chromatin fraction. Proteins in each fraction were resolved by SDS-polyacrylamide gel electrophoresis and transferred to a polyvinylidene difluoride membrane (Millipore, Billerica, MA). Immunoblot analysis was performed with antibodies to H-Ras (sc-520; Santa Cruz Biotechnology, Santa Cruz, CA), to Erk1/2 (9102; Cell Signaling Technology, Beverly, MA), to phosphorylated Erk1/2 (9101; Cell Signaling Technology), to Suz12 (ab12073; Abcam, Cambridge, MA), to α-tubulin (T5168; Sigma), to H3K27me3 (07-449; Millipore), and to histone H3 (ab1791; Abcam). Immune complexes were detected with horseradish peroxidase–conjugated secondary antibodies and Super Signal West Dura Luminol/Enhancer Solution (Thermo Scientific, Rockford, IL). The chemiluminescence signals were quantitated with a digital imaging system (VersaDoc; Bio-Rad, Hercules, CA).

### RT-qPCR analysis

Total RNA was isolated from cells and purified with the use of an SV Total RNA Isolation System (Promega). It was then subjected to RT with the use of a PrimeScript RT reagent kit (Takara Bio, Shiga, Japan) followed by real-time PCR analysis with a StepOnePlus Real Time PCR System (Life Technologies) and Fast SYBR Green Master Mix (Life Technologies). Data were analyzed according to the 2^−ΔΔCt^ method and were normalized by the amount of acidic ribosomal phosphoprotein P0 (Arbp) mRNA. The sequences and gene information of PCR primers are listed in Table S2.

### ChIP analysis

Cells were fixed with 0.6 or 1.0% formaldehyde for 10 or 5 min, respectively, at room temperature, after which glycine was added to the medium. The cells were then lysed and stored at −80°C until analysis. The lysates were thawed and subjected to ultrasonic treatment with the use of a Bioruptor (Diagenode, Denville, NJ) or Covaris S2 (Covaris, Woburn, MA) instrument in order to obtain chromatin fragments of 200 to 700 bp.

Antibodies to H3K27me3 (07-449; Millipore), to H3K9me2 (ab1220; Abcam), to H3K9me3 (ab8898; Abcam), or to H3 (ab1791; Abcam), or normal mouse (sc2025; Santa Cruz Biotechnology) or rabbit (sc2027; Santa Cruz Biotechnology) immunoglobulin G, were incubated with Protein A Dynabeads or Protein G Dynabeads (Life Technologies) to allow formation of bead-antibody complexes. Chromatin fragments were then subjected to immunoprecipitation with the bead-antibody complexes, after which the beads were washed and immunoprecipitated chromatin fragments were eluted and treated with RNase A and proteinase K. DNA was extracted from the samples with phenol-chloroform and was then precipitated with ethanol and dissolved in TE buffer.

Quantitative PCR analysis of ChIP DNA was performed as described above. Primer sequences and positions are listed in Table S3. Data were analyzed according to the 2^−(Ct of IP sample – Ct of input sample)^ method and are presented as a percentage of input.

### ChIP-seq and RNA-seq

For comprehensive analysis of Ras-dependent changes in gene expression and H3K27me content, we performed RNA-seq and ChIP-seq analyses at various times after Ras induction. We sampled cells at 0, 2, 4, 7, and 12 days after infection with the H-Ras(G12V) retroviral vector. We sequenced five and six samples for analysis of gene expression and H3K27me3, respectively. ChIP-seq libraries were prepared from ∼40 ng each of ChIP and input DNA with the use of a TruSeq DNA LT Sample Prep Kit (Illumina, San Diego, CA). RNA-seq libraries were prepared from 2 µg of total RNA with the use of a TruSeq RNA Sample Prep Kit v2 (Illumina). Two flow cells (16 lanes) of an Illumina HiSeq 2000 instrument were used. Libraries were clonally amplified in a flow cell and sequenced with the use of HiSeq Control Software 1.5 (Illumina) and a 48-nucleotide paired-end sequence. Image analysis and base calling were performed with the use of Real Time Analysis (RTA) 1.13 software. A total of 81,877,304 (RNA-seq) or 1,068,022,370 (ChIP-seq) reads was obtained per sample.

For SOLiD sequencing, ChIP-seq libraries were prepared from 20 ng each of ChIP and input samples with the use of a SOLiD Fragment Library Construction Kit with SizeSelect Gels (Life Technologies). For RNA-seq, total RNA (10 µg) was subjected to rRNA depletion (RiboMinus Eukaryote Kit for RNA-seq, Life Technologies) and RNA-seq library construction (SOLiD Whole Transcriptome Analysis Kit, Life Technologies). The libraries were clonally amplified on SOLiD P1 DNA Beads by emulsion PCR and sequenced with the SOLiD3Plus System (Life Technologies) to generate 50-base single-end reads.

Sequencing data of ChIP-seq and RNA-seq are available under the accession number of DRA001075 from DNA Data Bank of Japan Sequence Read Archive (DRA).

### Illumina sequence data analysis

FastQC (http://www.bioinformatics.babraham.ac.uk/projects/fastqc) analysis revealed low sequence quality for the last 4 bases of the second read of paired-end reads, and so these bases were trimmed before data analysis. For the sequence data analysis, UCSC mm9 and RefSeq were used as the reference mouse genome and gene model, respectively.

For gene expression analysis, paired-end reads were mapped to the mouse genome with the use of TopHat (ver. 2.0.8) [54]. Cufflinks (ver. 2.0.10) [55] was used to estimate gene expression level on the basis of fragments per kilobase of exon model per million mapped fragments (FPKM). Gene expression level was compared between control (Ras0) cells and Ras cells at 2, 4, 7, or 12 days after activation of Ras signaling with the use of Cuffdiff (ver. 2.0.10). A Q-value of <0.05 was set as a threshold for differential expression, resulting in the extraction of 933 genes as differentially expressed genes.

For H3K27me3 analysis, sequenced reads were mapped to the mouse genome with the use of bwa (ver. 0.5.9) [56]. Paired reads that were uniquely mapped to the genome were extracted. This filtering process discarded 174,644,447 (16.72%) reads per sample, and the remaining 870,116,495 (83.28%) reads were used for subsequent analyses. The sequence depth of our ChIP-seq data set was 40 (including insert), with 90% of bases in the genome being covered by at least one read. As far as we are aware, this is one of the most deeply sequenced histone modification marks to date.

For the purposes of our study, we defined the gene body as the genomic region from the TSS to the TTS. Introns are thus included in the gene body as well as exons. To compare methylation signals associated with genes of different sizes, we calculated the relative position of bins in the gene body as follows:
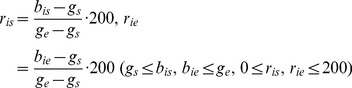
(1)where *g_s_* and *g_e_* represent the TSS and TTS of gene *g*, *b_is_* and *b_ie_* are the start and end positions of the *i*th bin in gene *g*, and *r_is_* and *r_ie_* are the normalized *i*th bin positions, respectively. The same normalization procedure was applied to the upstream and downstream regions of each gene, resulting in 600 data points per gene. The methylation signal associated with each bin was then assigned to the normalized positions. The mean and standard deviation of the methylation signal for the 600 data points obtained for each gene were calculated. The methylation signal around the TSS (TSS ±2.5 kb) was analyzed in the same manner.

To examine the methylation pattern across the gene body and adjacent regions, we randomly selected 2000 out of 23,232 RefSeq genes. The methylation signal of each selected gene was normalized so that the mean and standard deviation of the signal were equal to 0 and 1, respectively. A hierarchical clustering was performed according to the normalized methylation signal, and the results were visualized as a heat map in which the intensity of the methylation signal was color coded. Gene expression level was also displayed in the heat map (Figure 2B).

The relation between methylation and gene expression was investigated by comparison of the average methylation signal and FPKM. For this purpose, the average methylation signal in the gene body and in the region around the TSS (defined here as the region from −2.5 to +2.5 kb relative to the TSS) was calculated for each gene at all time points. Gene expression level was categorized into five classes including unexpressed genes (FPKM = 0), and the distribution of the methylation signal in each class is presented as a box-and-whisker plot (Figure 2E).

The average methylation signal in control (Ras0) cells compared with cells at various times after infection with the H-Ras(G12V) retroviral vector as well as the fold change in the methylation signal were calculated. If the latter fold change was ≥2, then the gene was extracted as a differentially methylated gene. A total of 1027 genes (gene body) or 1230 genes (region around the TSS) fulfilled this criterion, and these genes were further examined by comparison with differentially expressed genes. To examine changes in methylation over time, we performed a hierarchical clustering according to average methylation signal as described above (Figure 3B, Figure S2B).

To examine whether gene expression might be causally related to a change in H3K27me3 level, we defined and calculated “t-half” as shown in Figure S1C. Two such values were calculated for each gene, one for gene expression and the other for H3K27me3 level.

### SOLiD sequence data analysis

We obtained 130,133,653, 150,914,422, 226,490,377, and 250,941,002 reads from control (Vec) and Ras cells for RNA-seq and from Vec and Ras cells for ChIP-seq, respectively. Sequenced reads were mapped to the mouse genome with the use of the BioScope Map Data program (ver. 1.2). The following analyses were based on the mapping results. Gene expression level was estimated by calculating reads per kilobase of exon model per million mapped reads (RPKM) [57]. Overall gene expression level in Vec and Ras cells was normalized by the expression level of *Arbp*. To calculate the amount of H3K27me3, we split the mouse genome into 1-kb bins and used the number of reads in each bin as the raw methylation signal. This raw signal was normalized as Vec and Ras cells have the same number of reads and was then smoothed with the lowess function. Obtained signals were converted into wiggle (WIG) format and uploaded to the UCSC Genome Browser for visualization.

### Detection of novel transcripts

To identify putative novel transcripts, we mapped sequenced reads to the mouse genome with the use of TopHat (ver. 1.3.3). Given that our SOLiD sequence data included strand information, mapped reads on the Watson and Crick strand were analyzed separately. The number of mapped reads at each genome coordinate was converted to bigWig format and uploaded to the UCSC Genome Browser. Those regions that did not overlap with a RefSeq gene and showed a difference in expression level between Vec and Ras cells were manually inspected.

## Supporting Information

Figure S1 Hierarchical clustering of Ras-induced changes in gene transcription and calculation of t-half. (**A**) Number of genes whose expression was found to be affected by Ras signaling. (**B**) Clustering of time course profiles for Ras-induced changes in gene transcription. Each line represents one of the 933 genes whose transcription is regulated by Ras. The genes are divided into two groups shown in red or blue corresponding to a Ras-induced increase or decrease, respectively, in FPKM value. These two clusters are the source of the expression data in Figure 3B and Figure S2B. (**C**) Definition of “t-half” as the time corresponding to half of the difference between the H3K27me3 or transcription levels for Ras0 cells and cells expressing H-Ras(G12V) for 12 days.
(TIF)

Figure S2 Comprehensive analysis of Ras-induced changes in gene transcription and H3K27me3 content in the region around the TSS. (**A**) Venn diagram indicating the number of genes showing Ras-induced changes in expression and in the mean H3K27me3 level in the region around the TSS. (**B**) Clustering of the temporal profiles of mean H3K27me3 level in the region around the TSS. Each line represents one of 196 genes whose H3K27me3 level in the region around the TSS and expression changed in NIH 3T3 cells during expression of H-Ras(G12V) for the indicated times. Results of hierarchical clustering are depicted on the left with colors of purple, brown, and gray. Changes in expression level (FPKM) of individual genes (as determined in Figure S1B) are depicted on the right with colors of red (increase) or blue (decrease). (**C**) Averaged changes in expression and H3K27me3 level for the purple cluster (upper) and the gray cluster (lower) of genes shown in (B). Dashed lines represent t-half. (**D**) The t-half values for expression and mean H3K27me3 level in the region around the TSS for the purple and gray clusters in (B).
(TIF)

Figure S3 Additional examples of genes showing immediate changes in expression and delayed changes in H3K27me3 level induced by Ras signaling. (**A**) Time course of changes in H3K27me3 level at the *Plekha4*, *Ephx1*, *Bpifc*, and *Sorcs2* loci as determined by ChIP-seq analysis of Ras0 cells and cells infected with the retroviral vector for H-Ras(G12V) for 2, 4, 7, or 12 days. The regions for which the mean H3K27me3 level and corresponding t-half were calculated are highlighted in pink. Arrowheads indicate the regions of the genes analyzed by ChIP-qPCR in Figures S7A, S7C and S8B. (**B**) Changes in gene expression (FPKM) and mean H3K27me3 level for *Plekha4*, *Ephx1*, *Bpifc*, and *Sorcs2*. The t-half values are indicated by the dashed lines.
(TIF)

Figure S4 Validation of changes in H3K27me3 content of the gene body and in gene expression induced by Ras signaling. (**A**) SOLiD sequencing analysis of H3K27me3 level and gene expression at the *Itgb5* locus in Ras and Vec cells. The region of increased H3K27me3 level in Ras cells is highlighted in pink. Gene expression is presented as reads per kilobase of exon model per million mapped reads (RPKM). (**B**) RT-qPCR analysis of *Itgb5* expression in Ras cells relative to that in Vec cells. Data are representative of five independent experiments. (**C** and **D**) ChIP-qPCR analysis of H3K27me3 (C) and total H3 (D) for the gene body and flanking regions of *Itgb5* in Vec and Ras cells. Lowercase letters correspond to the positions indicated in (A). The amount of immunoprecipitated DNA is expressed as a percentage of input DNA. Data are representative of five independent experiments. (**E**) SOLiD sequencing analysis of H3K27me3 level and gene expression at the *Adcy7* locus in Ras and Vec cells. The region of decreased H3K27me3 level in Ras cells is highlighted in pink. (**F**) RT-qPCR analysis of *Adcy7* expression in Ras cells relative to that in Vec cells. Data are representative of five independent experiments. (**G** and **H**) ChIP-qPCR analysis of H3K27me3 (G) and total H3 (H) for the gene body and flanking regions of *Adcy7* in Vec and Ras cells. Lowercase letters correspond to the positions indicated in (E). Data are representative of five independent experiments.
(TIF)

Figure S5 ChIP-qPCR analysis of H3K9me2, H3K9me3, and H3K27me3 in the gene body for *Itgb5* and *Adcy7*. Lowercase letters after the gene names correspond to the positions of *Itgb5* (**A**) and *Adcy7* (**B**) loci shown in Figure S4A and S4E, respectively. Data are expressed as fold enrichment for Ras cells relative to Vec cells and are means ± SE from three independent experiments.
(TIF)

Figure S6 Knockdown efficiency and expression of *Itgb5* and *Adcy7* in cells depleted of Suz12 with different siRNA constructs. (**A**) Distribution of H3K27me3 at the *Hoxa3* locus in control (Ras0) cells as revealed by ChIP-seq analysis. The region around the *Hoxa10* gene was highly enriched with H3K27me3, whereas that around *Hoxa3* contained only a low level of H3K27me3. (**B**) Knockdown efficiency with Suz12 siRNA #1 in control cells as revealed by RT-qPCR analysis of *Suz12* mRNA. (**C**) Verification of H3K27me3 depletion with Suz12 siRNA #1 in control cells by ChIP-qPCR analysis with primers targeted to the regions indicated by the arrows in (A). The H3K27me3/H3 ratio around *Hoxa10* had decreased to a value similar to that for the *Hoxa3* gene. (**D**) NIH 3T3–Raf-ER cells transfected with Suz12 (#2 or #3) or control siRNAs and treated with 4HT or ethanol as in Figure 5A were subjected to immunoblot analysis. (**E** and **F**) The cells in (D) were also subjected to RT-qPCR analysis of relative *Suz12* (E) or *Itgb5* and *Adcy7* (F) expression. Data are representative of two independent experiments.
(TIF)

Figure S7 Raf-induced changes in H3K27me3 level at the gene body are not required for those in gene transcription. (**A**) RT-qPCR analysis of gene expression and ChIP-qPCR analysis of the ratio of H3K27me3 to total H3 at gene bodies for the indicated genes at the indicated times after exposure of NIH 3T3–Raf-ER cells to 4HT. The regions of the genes analyzed by ChIP-qPCR are indicated by the arrowheads in Figure S3. Data are expressed relative to the values for time 0 and are representative of four independent experiments. (**B**) RT-qPCR analysis of relative gene expression for NIH 3T3–Raf-ER cells transfected with Suz12 or control siRNAs and exposed to 4HT or ethanol (EtOH) vehicle as in Figure 5A. Data are means ± SE from three independent experiments. (**C**) ChIP-qPCR analysis of H3K27me3 normalized by total H3 at the gene bodies of the indicated genes for cells treated as in (B). Data are means ± SE from two independent experiments.
(TIF)

Figure S8 Additional examples of the effects of Ras signal inactivation on gene expression and H3K27me3 level in NIH 3T3–ER-Ras cells. NIH 3T3–ER-Ras cells were exposed to 4HT for 9 days and then incubated in the absence of 4HT as in Figure 6A. The cells were subjected to RT-qPCR analysis (**A**) of relative *Plekha4*, *Ephx1*, *Bpifc*, and *Sorcs2* expression as well as to ChIP-qPCR analysis (**B**) of H3K27me3 and total H3 at the regions of the genes indicated by the arrowheads in Figure S3. Data are means ± SE from two independent experiments.
(TIF)

Figure S9 Identification of novel transcripts derived from an additional intergenic region subject to Ras-induced modulation of H3K27me3 content. (**A**) H3K27me3 ChIP-seq results as well as strand-specific assignment of sequencing reads from RNA-seq analysis for the *Il33* locus in Vec and Ras cells. The intergenic region showing a decrease in H3K27me3 content in response to Ras signaling is highlighted in pink. Antisense transcription from the region upstream of *Il33* (*uIl33*) was observed predominantly in Ras cells, with the predicted transcribed region being indicated by the magenta box. Visual inspection suggests that *uIl33* might contribute to a novel transcriptional variant of an upstream annotated gene (9930021J03Rik in RefSeq). (**B**) RT-qPCR analysis of expression as well as ChIP-qPCR analysis of H3K27me3 normalized by total H3 for *uIl33* at the indicated times after exposure of NIH 3T3 cells expressing Raf-ER to 4HT. PCR was performed with primer sets targeted to the positions a and b indicated in (A).
(TIF)

Figure S10 Effects of Ras signaling on expression of genes for H3K27me3-related enzymes. (**A**) Expression of genes for major PRC2 components in Ras and Vec cells. Gene expression is presented as RPKM as determined by SOLiD sequencing. (**B** and **C**) NIH 3T3–Raf-ER cells transfected with Bmi1 (B), Jmjd3 (C), or control siRNAs and treated with 4HT or ethanol vehicle as in Figure 5A were subjected to RT-qPCR analysis of relative expression of *Bmi1*, *Itgb5*, and *Smad6* (B) or of *Jmjd3* and *Adcy7* (C). Data are means ± SE from three independent experiments.
(TIF)

Table S1 Illumina sequencing information. Sequencing throughput and filtering summary of RNA-seq and ChIP-seq analyses of samples at various times after Ras induction.
(XLSX)

Table S2 Primers for RT-qPCR analysis. List of all primer sequences (5′ to 3′) used for RT-qPCR and gene information in this study.
(XLSX)

Table S3 Primers for ChIP-qPCR analysis. List of all primer sequences used for ChIP-qPCR. We represent primer sequences (5′ to 3′) and primer position in mouse reference genome (NCBI37/mm9).
(XLSX)
