# Supplementary figures and images for: Ras-Induced Changes in H3K27me3 Occur after Those in Transcriptional Activity
Source: PLoS Genet. 2013 Aug 29;9(8):e1003698. doi: 10.1371/journal.pgen.1003698 (PMC3757056; doi:10.1371/journal.pgen.1003698)

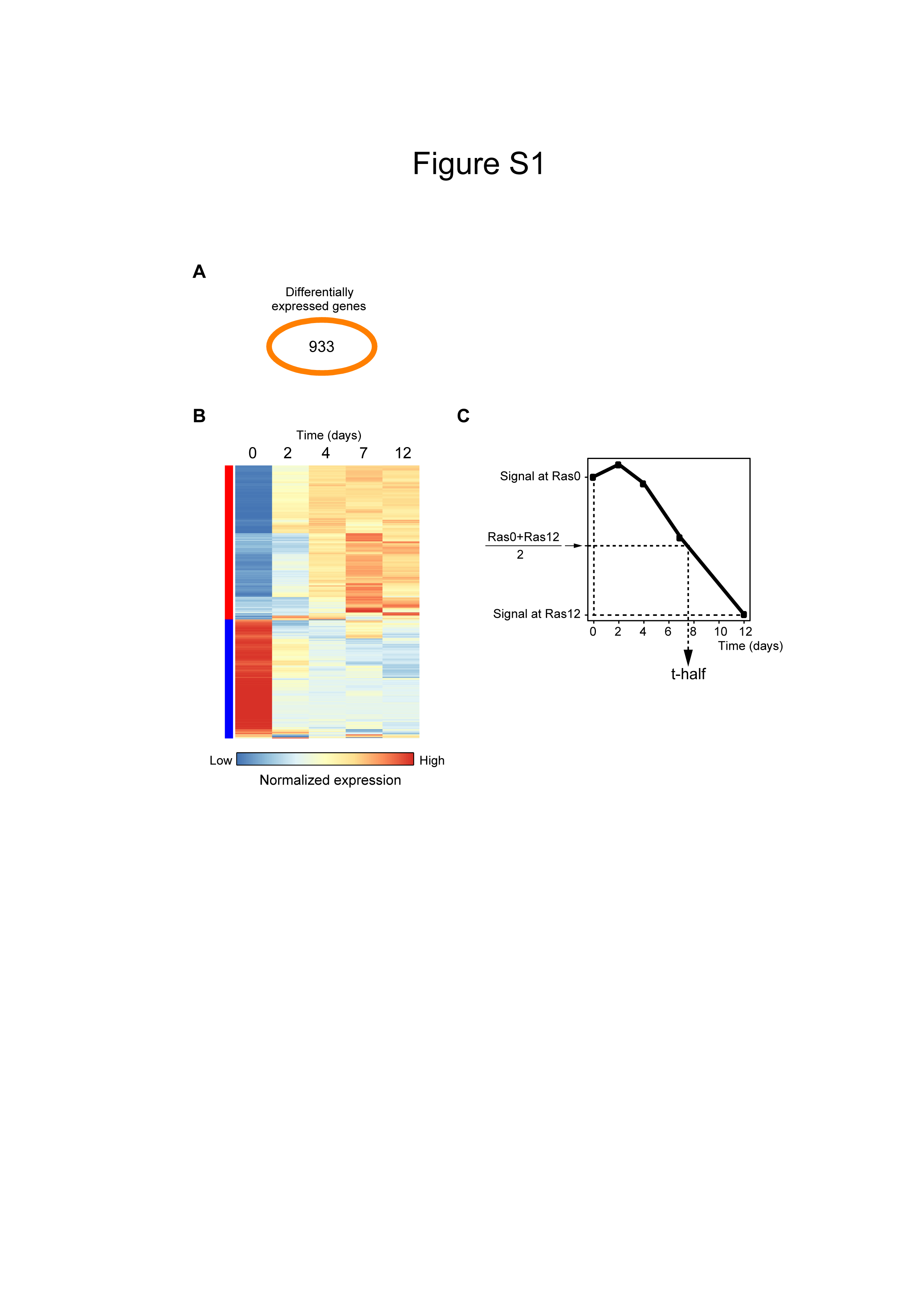

Supplement: Figure S1 — Hierarchical clustering of Ras-induced changes in gene transcription and calculation of t-half. (A) Number of genes whose expression was found to be affected by Ras signaling. (B) Clustering of time course profiles for Ras-induced changes in gene transcription. Each line represents one of the 933 genes whose transcription is regulated by Ras. The genes are divided into two groups shown in red or blue corresponding to a Ras-induced increase or decrease, respectively, in FPKM value. These two clusters are the source of the expression data in Figure 3B and Figure S2B. (C) Definition of “t-half” as the time corresponding to half of the difference between the H3K27me3 or transcription levels for Ras0 cells and cells expressing H-Ras(G12V) for 12 days. (TIF) [file pgen.1003698.s001.tif]

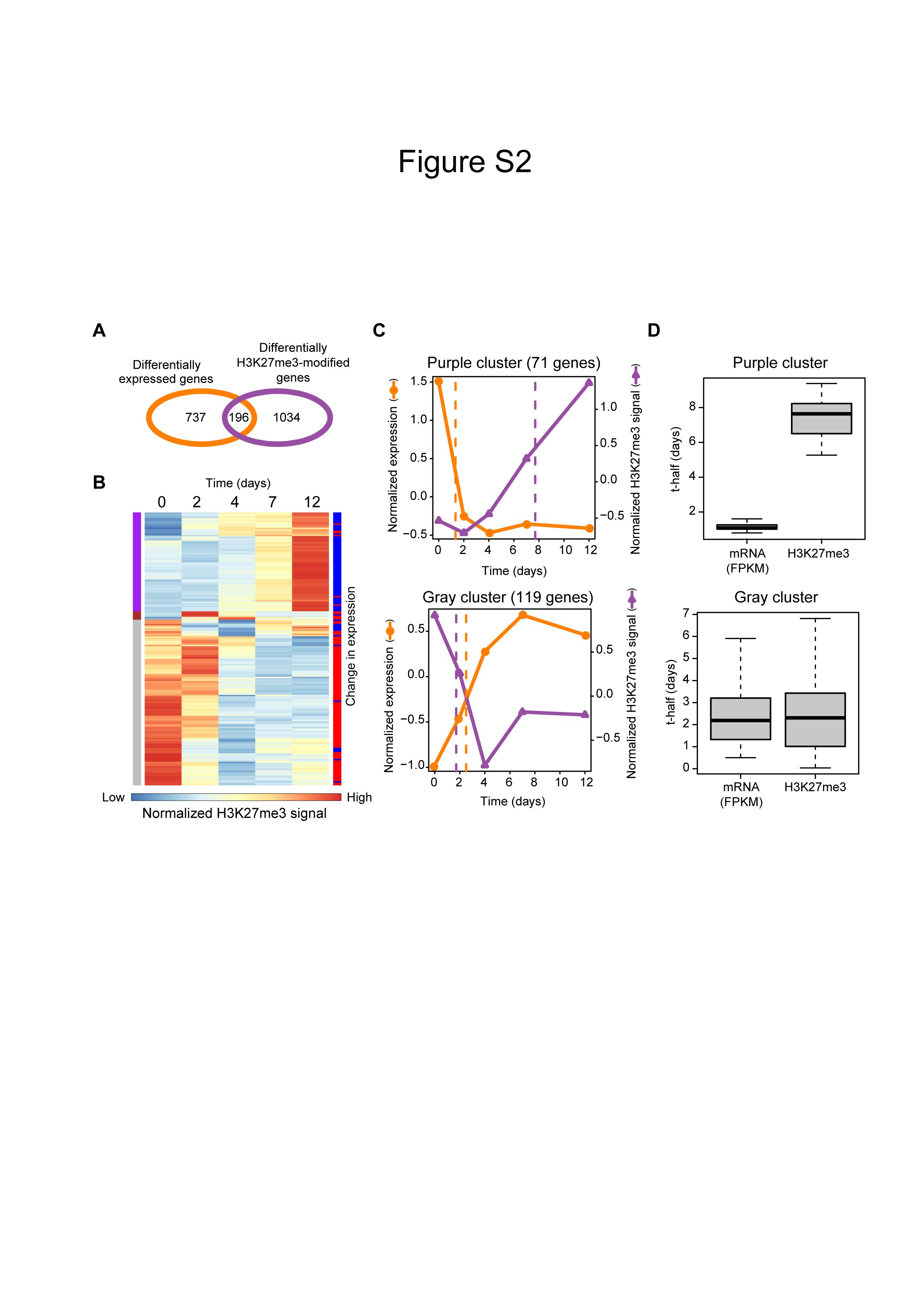

Supplement: Figure S2 — Comprehensive analysis of Ras-induced changes in gene transcription and H3K27me3 content in the region around the TSS. (A) Venn diagram indicating the number of genes showing Ras-induced changes in expression and in the mean H3K27me3 level in the region around the TSS. (B) Clustering of the temporal profiles of mean H3K27me3 level in the region around the TSS. Each line represents one of 196 genes whose H3K27me3 level in the region around the TSS and expression changed in NIH 3T3 cells during expression of H-Ras(G12V) for the indicated times. Results of hierarchical clustering are depicted on the left with colors of purple, brown, and gray. Changes in expression level (FPKM) of individual genes (as determined in Figure S1B) are depicted on the right with colors of red (increase) or blue (decrease). (C) Averaged changes in expression and H3K27me3 level for the purple cluster (upper) and the gray cluster (lower) of genes shown in (B). Dashed lines represent t-half. (D) The t-half values for expression and mean H3K27me3 level in the region around the TSS for the purple and gray clusters in (B). (TIF) [file pgen.1003698.s002.tif]

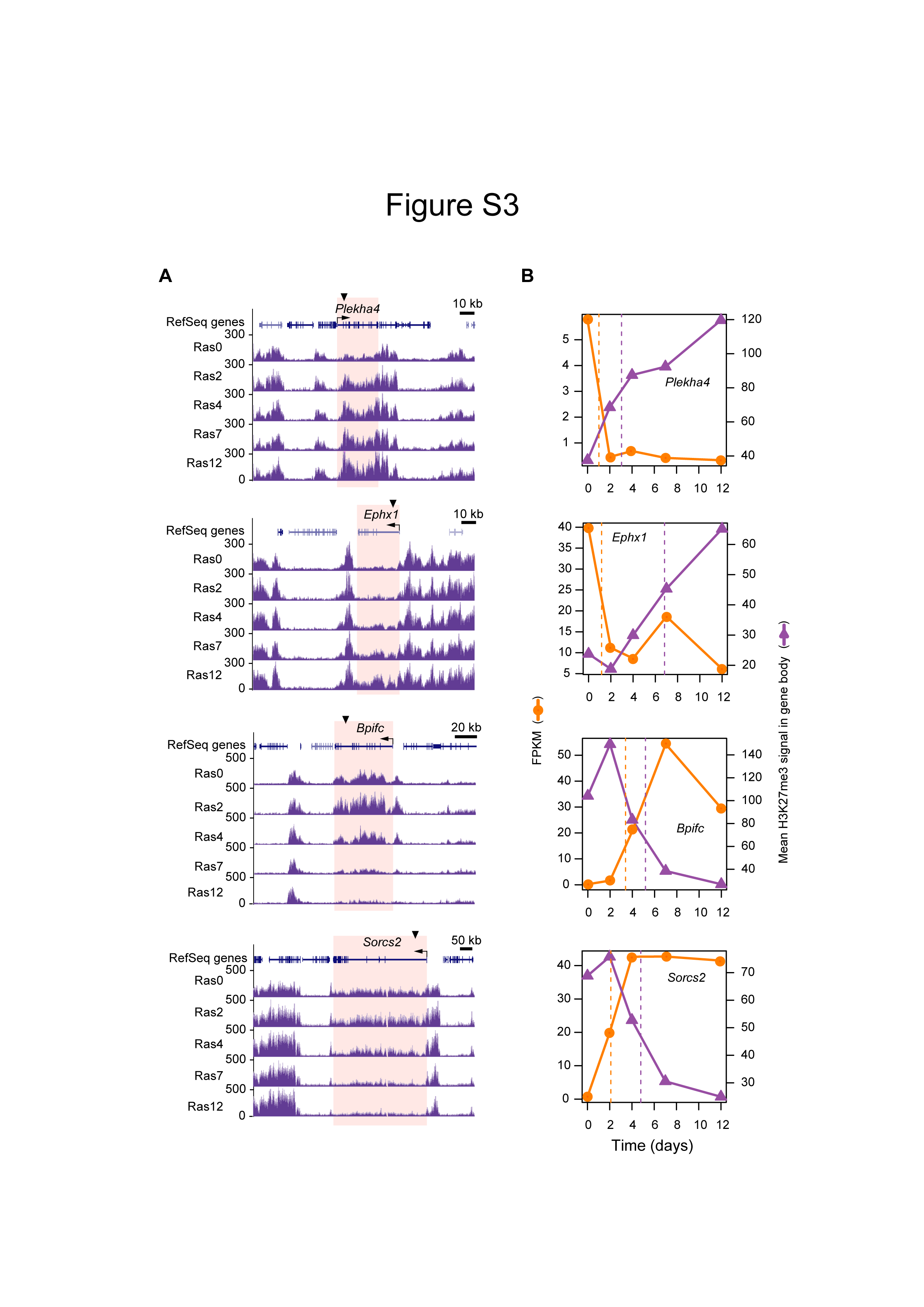

Supplement: Figure S3 — Additional examples of genes showing immediate changes in expression and delayed changes in H3K27me3 level induced by Ras signaling. (A) Time course of changes in H3K27me3 level at the Plekha4, Ephx1, Bpifc, and Sorcs2 loci as determined by ChIP-seq analysis of Ras0 cells and cells infected with the retroviral vector for H-Ras(G12V) for 2, 4, 7, or 12 days. The regions for which the mean H3K27me3 level and corresponding t-half were calculated are highlighted in pink. Arrowheads indicate the regions of the genes analyzed by ChIP-qPCR in Figures S7A, S7C and S8B. (B) Changes in gene expression (FPKM) and mean H3K27me3 level for Plekha4, Ephx1, Bpifc, and Sorcs2. The t-half values are indicated by the dashed lines. (TIF) [file pgen.1003698.s003.tif]

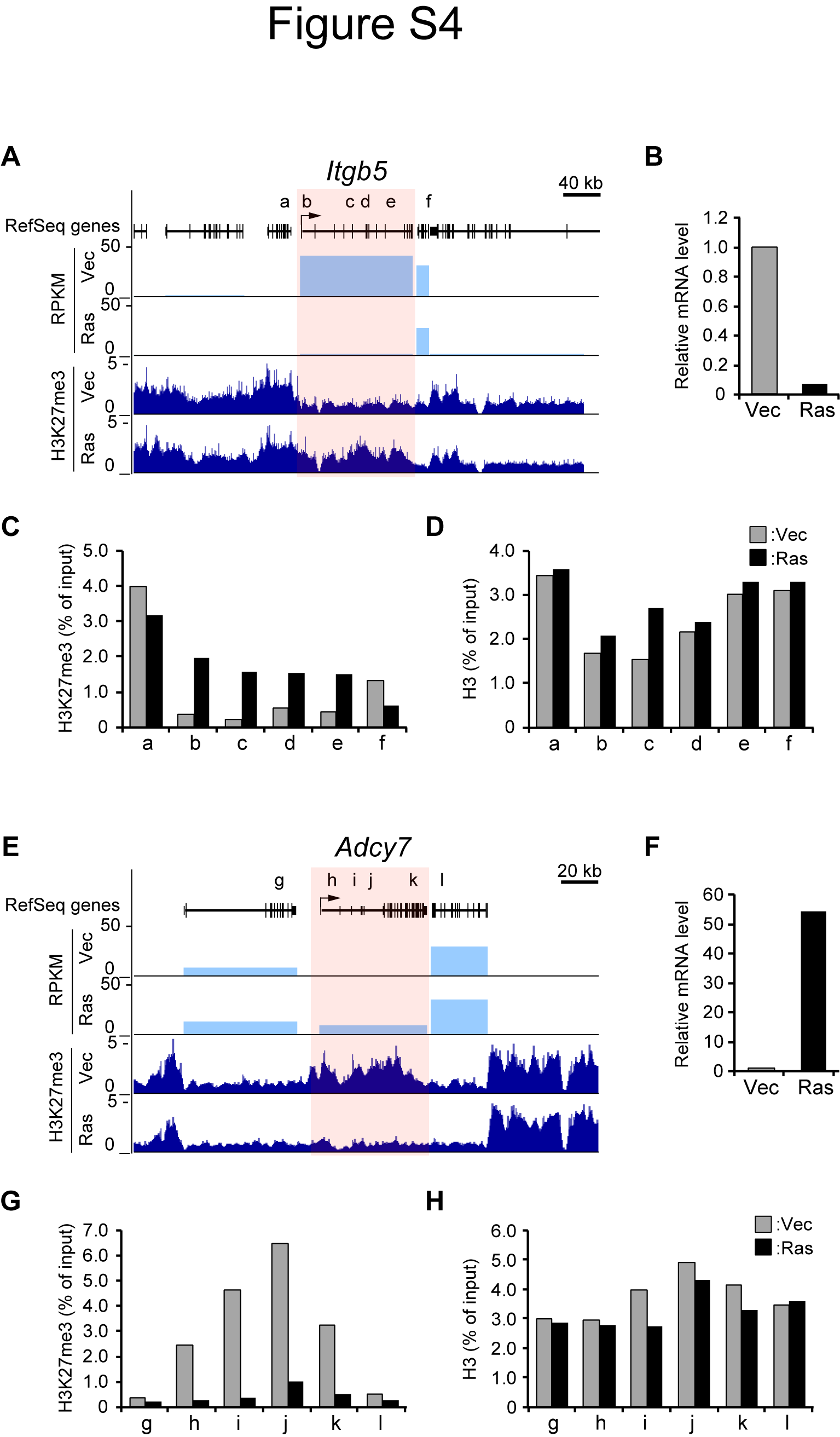

Supplement: Figure S4 — Validation of changes in H3K27me3 content of the gene body and in gene expression induced by Ras signaling. (A) SOLiD sequencing analysis of H3K27me3 level and gene expression at the Itgb5 locus in Ras and Vec cells. The region of increased H3K27me3 level in Ras cells is highlighted in pink. Gene expression is presented as reads per kilobase of exon model per million mapped reads (RPKM). (B) RT-qPCR analysis of Itgb5 expression in Ras cells relative to that in Vec cells. Data are representative of five independent experiments. (C and D) ChIP-qPCR analysis of H3K27me3 (C) and total H3 (D) for the gene body and flanking regions of Itgb5 in Vec and Ras cells. Lowercase letters correspond to the positions indicated in (A). The amount of immunoprecipitated DNA is expressed as a percentage of input DNA. Data are representative of five independent experiments. (E) SOLiD sequencing analysis of H3K27me3 level and gene expression at the Adcy7 locus in Ras and Vec cells. The region of decreased H3K27me3 level in Ras cells is highlighted in pink. (F) RT-qPCR analysis of Adcy7 expression in Ras cells relative to that in Vec cells. Data are representative of five independent experiments. (G and H) ChIP-qPCR analysis of H3K27me3 (G) and total H3 (H) for the gene body and flanking regions of Adcy7 in Vec and Ras cells. Lowercase letters correspond to the positions indicated in (E). Data are representative of five independent experiments. (TIF) [file pgen.1003698.s004.tif]

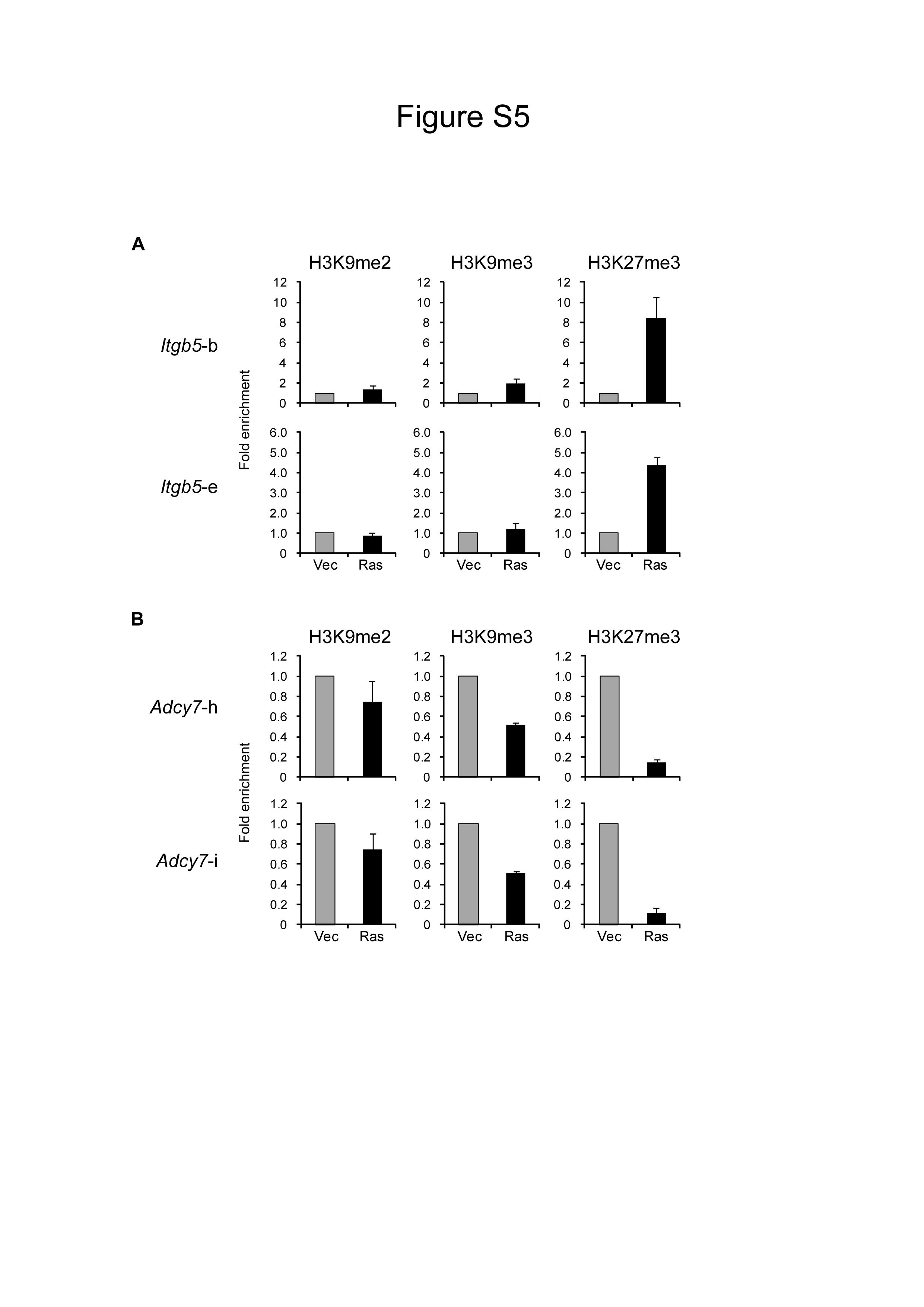

Supplement: Figure S5 — ChIP-qPCR analysis of H3K9me2, H3K9me3, and H3K27me3 in the gene body for Itgb5 and Adcy7. Lowercase letters after the gene names correspond to the positions of Itgb5 (A) and Adcy7 (B) loci shown in Figure S4A and S4E, respectively. Data are expressed as fold enrichment for Ras cells relative to Vec cells and are means ± SE from three independent experiments. (TIF) [file pgen.1003698.s005.tif]

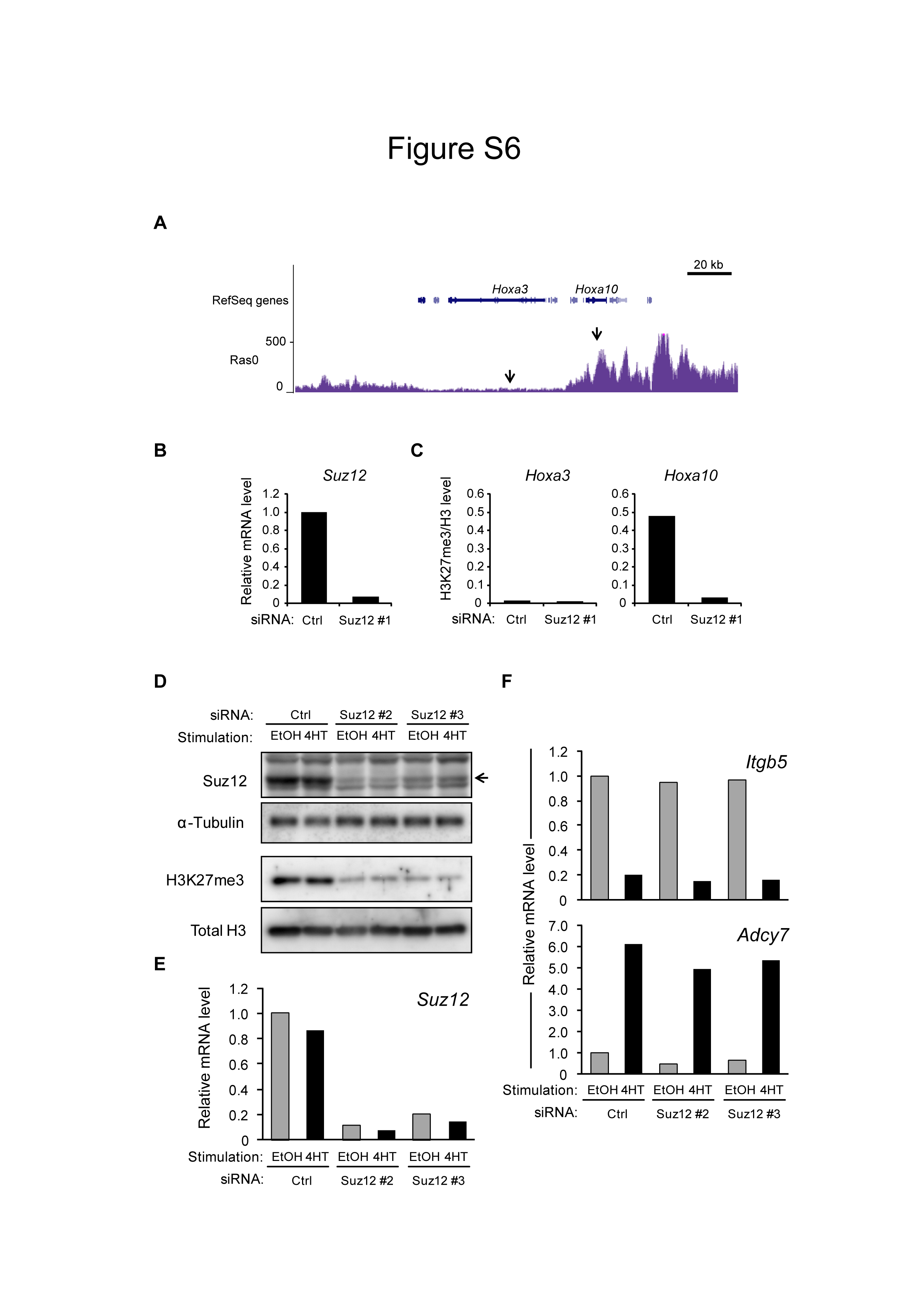

Supplement: Figure S6 — Knockdown efficiency and expression of Itgb5 and Adcy7 in cells depleted of Suz12 with different siRNA constructs. (A) Distribution of H3K27me3 at the Hoxa3 locus in control (Ras0) cells as revealed by ChIP-seq analysis. The region around the Hoxa10 gene was highly enriched with H3K27me3, whereas that around Hoxa3 contained only a low level of H3K27me3. (B) Knockdown efficiency with Suz12 siRNA #1 in control cells as revealed by RT-qPCR analysis of Suz12 mRNA. (C) Verification of H3K27me3 depletion with Suz12 siRNA #1 in control cells by ChIP-qPCR analysis with primers targeted to the regions indicated by the arrows in (A). The H3K27me3/H3 ratio around Hoxa10 had decreased to a value similar to that for the Hoxa3 gene. (D) NIH 3T3–Raf-ER cells transfected with Suz12 (#2 or #3) or control siRNAs and treated with 4HT or ethanol as in Figure 5A were subjected to immunoblot analysis. (E and F) The cells in (D) were also subjected to RT-qPCR analysis of relative Suz12 (E) or Itgb5 and Adcy7 (F) expression. Data are representative of two independent experiments. (TIF) [file pgen.1003698.s006.tif]

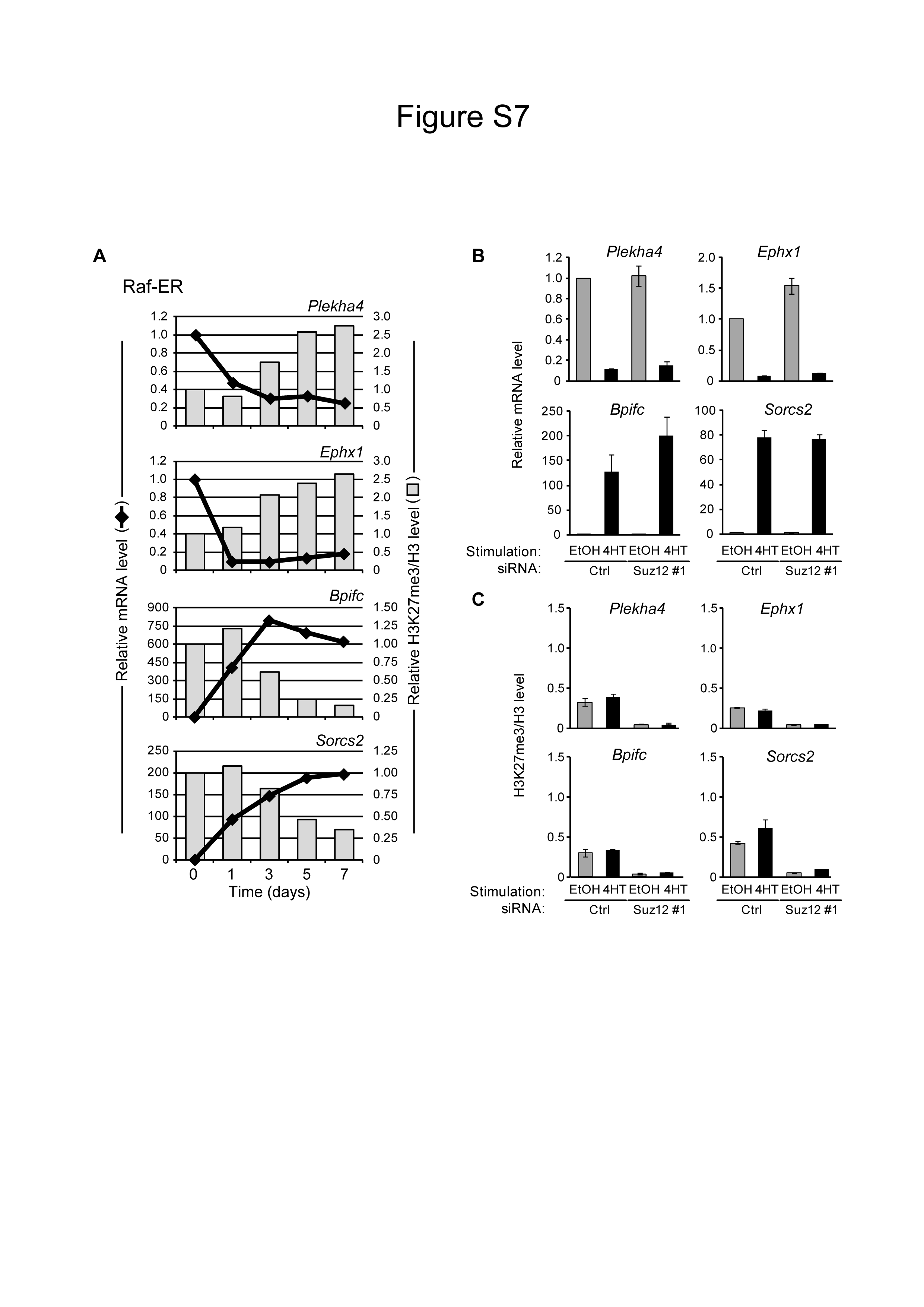

Supplement: Figure S7 — Raf-induced changes in H3K27me3 level at the gene body are not required for those in gene transcription. (A) RT-qPCR analysis of gene expression and ChIP-qPCR analysis of the ratio of H3K27me3 to total H3 at gene bodies for the indicated genes at the indicated times after exposure of NIH 3T3–Raf-ER cells to 4HT. The regions of the genes analyzed by ChIP-qPCR are indicated by the arrowheads in Figure S3. Data are expressed relative to the values for time 0 and are representative of four independent experiments. (B) RT-qPCR analysis of relative gene expression for NIH 3T3–Raf-ER cells transfected with Suz12 or control siRNAs and exposed to 4HT or ethanol (EtOH) vehicle as in Figure 5A. Data are means ± SE from three independent experiments. (C) ChIP-qPCR analysis of H3K27me3 normalized by total H3 at the gene bodies of the indicated genes for cells treated as in (B). Data are means ± SE from two independent experiments. (TIF) [file pgen.1003698.s007.tif]

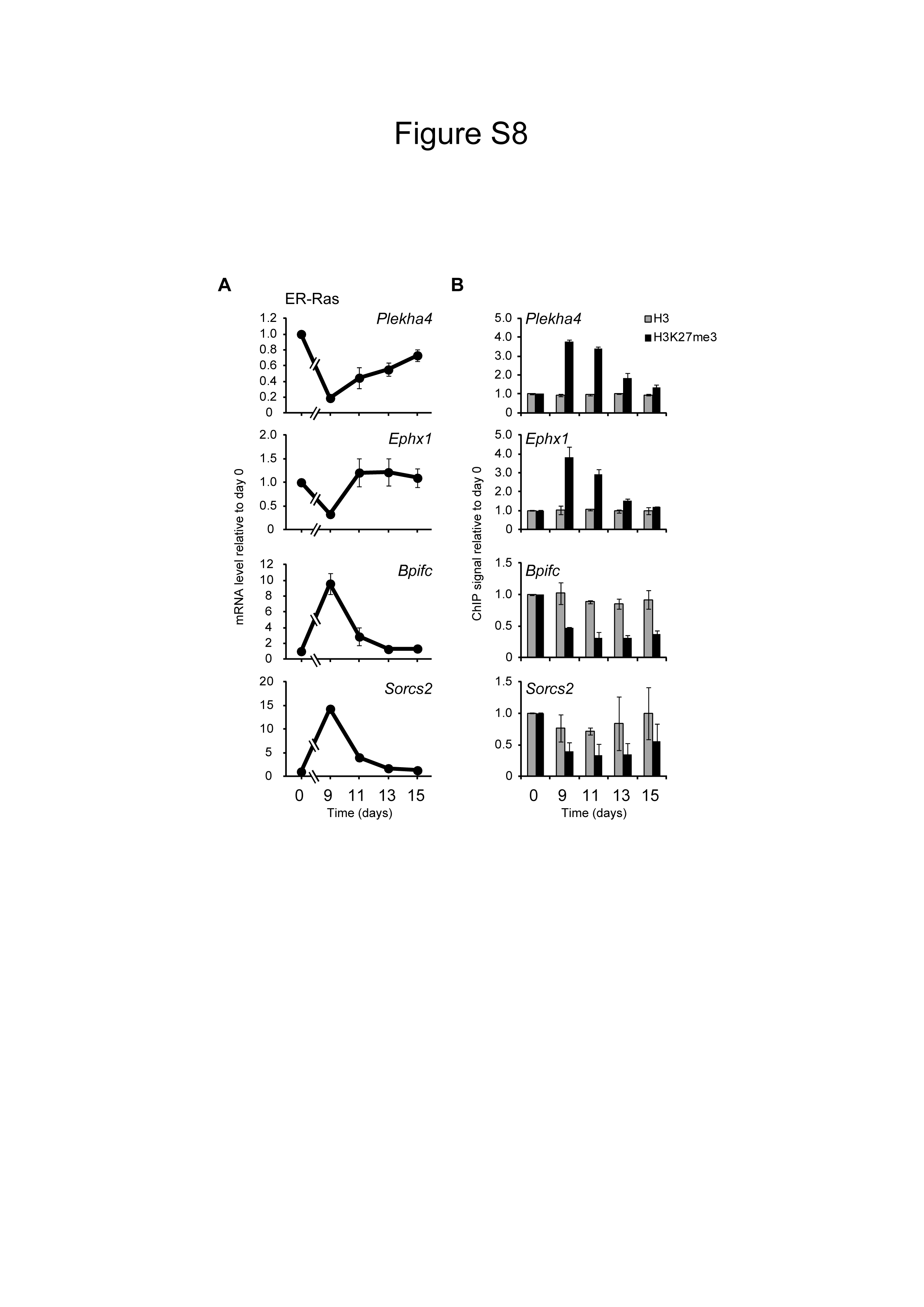

Supplement: Figure S8 — Additional examples of the effects of Ras signal inactivation on gene expression and H3K27me3 level in NIH 3T3–ER-Ras cells. NIH 3T3–ER-Ras cells were exposed to 4HT for 9 days and then incubated in the absence of 4HT as in Figure 6A. The cells were subjected to RT-qPCR analysis (A) of relative Plekha4, Ephx1, Bpifc, and Sorcs2 expression as well as to ChIP-qPCR analysis (B) of H3K27me3 and total H3 at the regions of the genes indicated by the arrowheads in Figure S3. Data are means ± SE from two independent experiments. (TIF) [file pgen.1003698.s008.tif]

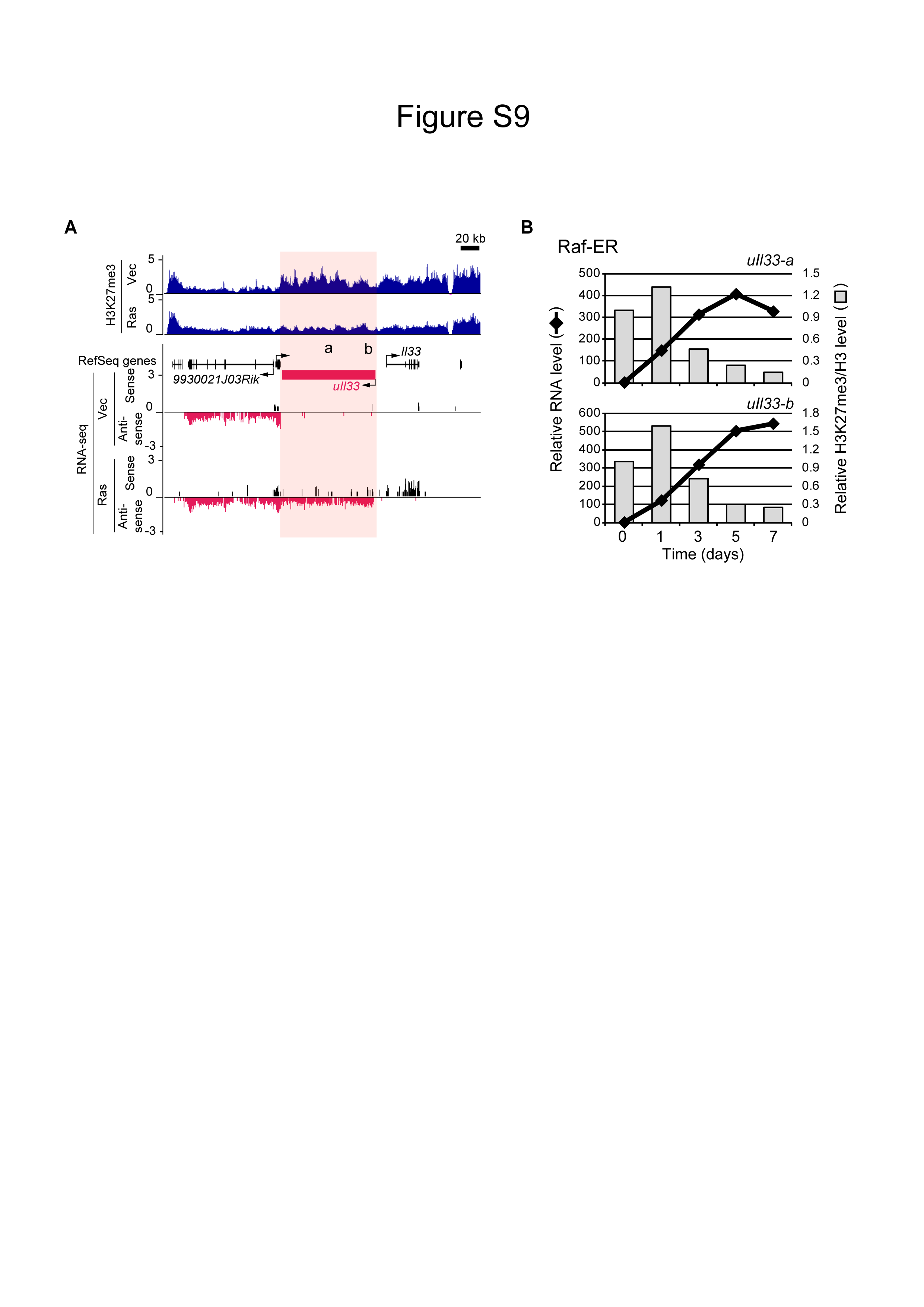

Supplement: Figure S9 — Identification of novel transcripts derived from an additional intergenic region subject to Ras-induced modulation of H3K27me3 content. (A) H3K27me3 ChIP-seq results as well as strand-specific assignment of sequencing reads from RNA-seq analysis for the Il33 locus in Vec and Ras cells. The intergenic region showing a decrease in H3K27me3 content in response to Ras signaling is highlighted in pink. Antisense transcription from the region upstream of Il33 (uIl33) was observed predominantly in Ras cells, with the predicted transcribed region being indicated by the magenta box. Visual inspection suggests that uIl33 might contribute to a novel transcriptional variant of an upstream annotated gene (9930021J03Rik in RefSeq). (B) RT-qPCR analysis of expression as well as ChIP-qPCR analysis of H3K27me3 normalized by total H3 for uIl33 at the indicated times after exposure of NIH 3T3 cells expressing Raf-ER to 4HT. PCR was performed with primer sets targeted to the positions a and b indicated in (A). (TIF) [file pgen.1003698.s009.tif]

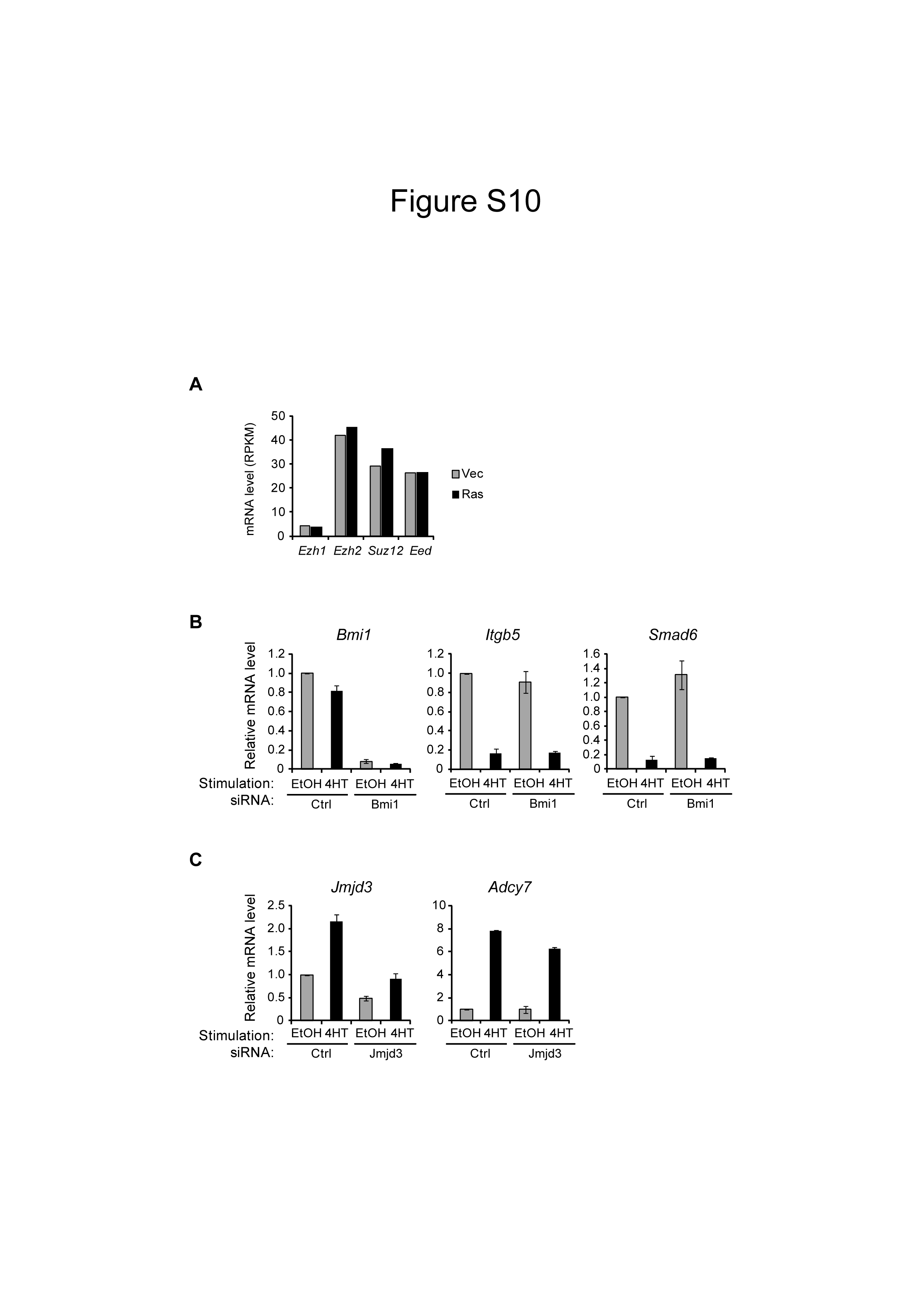

Supplement: Figure S10 — Effects of Ras signaling on expression of genes for H3K27me3-related enzymes. (A) Expression of genes for major PRC2 components in Ras and Vec cells. Gene expression is presented as RPKM as determined by SOLiD sequencing. (B and C) NIH 3T3–Raf-ER cells transfected with Bmi1 (B), Jmjd3 (C), or control siRNAs and treated with 4HT or ethanol vehicle as in Figure 5A were subjected to RT-qPCR analysis of relative expression of Bmi1, Itgb5, and Smad6 (B) or of Jmjd3 and Adcy7 (C). Data are means ± SE from three independent experiments. (TIF) [file pgen.1003698.s010.tif]
